# PEGylated Tween 80-functionalized chitosan-lipidic nano-vesicular hybrids for heightening nose-to-brain delivery and bioavailability of metoclopramide

**DOI:** 10.1080/10717544.2023.2189112

**Published:** 2023-03-14

**Authors:** Saeed A. S. Al-Zuhairy, Mahmoud H. Teaima, Nabil A. Shoman, Mohamed Elasaly, Mohamed A. El-Nabarawi, Hossam S. El-Sawy

**Affiliations:** a Department of Pharmacy, Kut University Collage, Kut, Iraq; b Department of Pharmaceutics and Industrial Pharmacy, Faculty of Pharmacy, Cairo University, Cairo, Egypt; c Department of Pharmaceutics and Pharmaceutical Technology, Faculty of Pharmacy, Ahram Canadian University, Giza, Egypt; d Pharmaceutical Inspection Department, Medical Service Sector, Ministry of Interior, Cairo, Egypt; e Department of Pharmaceutics and Pharmaceutical Technology, Faculty of Pharmacy, Egyptian Russian University, Cairo, Egypt

**Keywords:** Lipidic-based nanovesicles, PEGylation, Tween 80 functionalization, optimization, nose-to-brain delivery, bioavailability

## Abstract

A PEGylated Tween 80–functionalized chitosan–lipidic (PEG-T-Chito-Lip) nano-vesicular hybrid was developed for intranasal administration as an alternative delivery route to help improve the poor oral bioavailability of BCS class-III model/antiemetic (metoclopramide hydrochloride; MTC). The influence of varying levels of chitosan, cholesterol, PEG 600, and Tween 80 on the stability/release parameters of the formulated nanovesicles was optimized using Draper-Lin Design. Two optimized formulations (Opti-Max and Opti-Min) with both maximized and minimized MTC-release goals, were predicted, characterized, and proved their vesicular outline *via* light/electron microscopy, along with the mutual prompt/extended *in-vitro* release patterns. The dual-optimized MTC–loaded PEG-T-Chito-Lip nanovesicles were loaded in intranasal *in-situ* gel (ISG) and further underwent *in-vivo* pharmacokinetics/nose-to-brain delivery valuation on Sprague-Dawley rats. The absorption profiles in plasma (plasma-AUC_0-∞_) of the intranasal dual-optimized MTC–loaded nano-vesicular ISG formulation in pretreated rats were 2.95-fold and 1.64-fold more than rats pretreated with orally administered MTC and intranasally administered raw MTC-loaded ISG formulation, respectively. Interestingly, the brain-AUC_0-∞_ of the intranasal dual-optimized MTC–loaded ISG was 10 and 3 times more than brain-AUC_0-∞_ of the MTC-oral tablet and the intranasal raw MTC-loaded ISG, respectively. It was also revealed that the intranasal dual-optimized ISG significantly had the lowest liver-AUC_0-∞_ (862.19 ng.g^−1^.h^−1^) versus the MTC-oral tablet (5732.17 ng.g^−1^.h^−1^) and the intranasal raw MTC-loaded ISG (1799.69 ng.g^−1^.h^−1^). The brain/blood ratio profile for the intranasal dual-optimized ISG was significantly enhanced over all other MTC formulations (P < 0.05). Moreover, the 198.55% drug targeting efficiency, 75.26% nose-to-brain direct transport percentage, and 4.06 drug targeting index of the dual-optimized formulation were significantly higher than those of the raw MTC-loaded ISG formulation. The performance of the dual-optimized PEG-T-Chito-Lip nano-vesicular hybrids for intranasal administration evidenced MTC-improved bioavailability, circumvented hepatic metabolism, and enhanced brain targetability, with increased potentiality in heightening the convenience and compliance for patients.

## 1. Introduction

The nasal route of administration is one of most promising approaches that has been widely investigated and proved its ability in enhancing drugs’ bioavailability, targeting delivery of medications to brain and bypassing hepatic first pass effect (Laffleur & Bauer, 2021). The blood-brain barrier (BBB) and the blood cerebrospinal fluid barrier can be crossed non-invasively by using the nasal route for nose-to-brain drug delivery. In addition to the nose’s proximity to the brain, the olfactory and trigeminal nerves in the nose are unique in that they connect directly to the brain without passing through the BBB’s limitations (Gänger & Schindowski, 2018). These mutual advantages can be potentially utilized for our drug model under investigation; Metoclopramide hydrochloride (MTC).

MTC has been classified as Biopharmaceutics Classification System (BCS) Class III; a high water-soluble drug with poor permeability, which explains the wide variability and poor bioavailability of orally administered MTC (Stosik et al., 2008). MTC also suffers from extensive hepatic metabolism, which significantly limits its efficiency (Lee & Kuo, 2010; Shakhatreh et al., 2019). However, MTC is one of the most renowned medications that widely prescribed for the treatment of nausea and vomiting. Regarding MTC site of action, the chemoreceptor trigger zone (CTZ) in the postrema region of the brain is where MTC mainly inhibits the dopamine D_2_ and serotonin 5-HT_3_ receptors, which are responsible for the antiemetic actions (Lee & Kuo, 2010). MTC is the sole FDA-approved antiemetic drug for the management of diabetic gastroparesis (Pasricha et al., 2006). The FDA clearly states that MTC is recommended for relieving symptoms in adults with acute and recurring diabetic gastroparesis and treating adults with gastroesophageal reflux symptoms (Shakhatreh et al., 2019). MTC also helps chemotherapy patients who are experiencing nausea and vomiting (Herrstedt et al., 2022).

Therefore, choosing a different delivery strategy could potentially have an effect on MTC bioavailability and consequently effectiveness, in addition to evading GIT degradation and first-pass effect. This strategy shall also reduce the possibility of MTC-related EPRs and other unfavorable side effects. The intranasal approach can both enhance MTC systemic bioavailability (for peripheral site of action), as well as the brain delivery of MTC (for central site of action) with much-reduced hepatic first pass effect. A previous study investigated the intranasal delivery of MTC *in-situ* gel (ISG) and revealed the enhanced systemic bioavailability (54.6%) compared with the measured oral bioavailability (40.7%) (Mahajan & Gattani, 2010). Zaki et al. reported in many research works the superiority of the nasal delivery route of the spray and ISG intranasal dosage forms over the oral dosage form in improving MTC systemic bioavailability (Zaki et al., 2007, 2006a, 2006b). However, the mentioned previous work did not take into consideration to assess brain delivery within their investigations. More sophisticated delivery systems could be utilized for better brain delivery and bioavailability.

Many delivery nano-systems are capable of carrying drugs to the CNS and crossing the BBB. They can be loaded into the intranasal dosage form for further augmented brain targetability and improved systemic bioavailability. By functionalization of these nanovesicles with polymers like polyethylene glycol (PEG) and/or Tween 80, a potential generation of nanovesicles is designed that can evade the reticuloendothelial system’s (RES) activity and, as a result, have longer half-lives. The phagocytic cells scarcely detect the so-coated nano-systems, resulting in a longer blood circulation (Claudio et al., 2016). Despite the longer half-life, these nanovesicles could not efficiently penetrate the BBB and reach target sites at a therapeutic concentration, even if they were stealthy. The production of positively charged nanovesicular hybrids (by integrating positively-charged polymers like chitosan) to promote the adsorption transcytosis pathway of nanovesicles through BBB is one method for increasing the transport of medications to the brain using nanovesicular delivery systems (Denora et al., 2009).

In conclusion, it is crucial to create/adapt strategies that deliver active moieties *via* a suitable delivery system, with increased efficacy and bioavailability, enhanced delivery to site of action and fewer unwanted side effects with better patient compliance and convenience. Consequently, we are inspecting the intranasal delivery of a dual-optimized MTC–loaded PEGylated Tween 80–functionalized chitosan–lipidic (PEG-T-Chito-Lip) nano-vesicular hybrids (with dual maximized and minimized MTC-release goals) that can bypass the hepatic first-pass metabolism and achieve heightened bioavailability and therapeutic efficacy with the optimized MTC concentrations (systemically and in the brain).

## 2. Experimental

### 2.1. Materials

Metoclopramide hydrochloride (MTC) was kindly gifted from Egyptian International Pharmaceutical Industries Company (EIPICO), Tenth of Ramadan City, Egypt. Potassium phosphotungstate was kindly supplied as a gift from National Research Center, Pharmaceutical Technology department, Cairo, Egypt. Cholesterol, Poly ethylene glycol 600 (PEG 600), Acetonitrile HPLC grade, and Chitosan of medium molecular weight (Sigma Aldrich, St. Louis, MO, USA). Tween 80 and Ethyl alcohol (99%) (El-Nasr Pharmaceutical Chemicals Co., Abuzaabal, Cairo, Egypt). Orthophosphoric acid and Span® 60 (Merck Schuchardit OHG, Hohenburnn, Germany). Carbopol 940, Acros Organics (Morris Plains, NJ, USA). Poloxamer 407 (Xi’an Lyphar Biotech Co., Ltd, Xi’an, China). Disodium hydrogen phosphate (Na_2_HPO_4_), Potassium dihydrogen orthophosphate (KH_2_PO_4_), and Sodium chloride (NaCl) (Oxford Lab Chem, Mumbai, India). Acetonitrile (Supelco, Bellefonte, PA, USA), Chloroform HPLC grade (Honeywell Riedel-de Haën, Seelze, Germany). HPLC grade water (Merck, Kenilworth, NJ, USA).

### 2.2. Design of experiment

To plan the experimental design for making MTC–loaded lipidic nano-vesicular hybrids, the Draper-Lin small composite design (DLD) method was employed. Four factors were selected to study their effects on the selected physical stability and release behavior attributes, namely; the mean vesicle size (Y_1_), zeta potential (Y_2_), entrapment efficiency (Y_3_), the percentage of MTC initial release after 1 h (Y_4_), and the percentage of MTC cumulative release after 24 h (Y_5_) (Abdelbary & Aboughaly, 2015; El-Say et al., 2015, 2016; Gajra et al., 2015; Solanki et al., 2007). These factors were the amount of chitosan per mg (X_1_), cholesterol molar ratio concentration (X_2_), PEG 600 and Tween 80 concentration percentage (X_3_ and X_4_, respectively). The objectives to be attained and the investigated dependent variables’ responses are stated in Table 1. In light of this, 19 various formulations were created in a fully randomized order, as shown in Table 2. Statgraphics^®^ Centurion 18 Software (StatPoint, Inc., Warrenton, VA, USA) was used to conduct the statistical study.

**Table 1.** Draper-Lin small composite design (DLD) independent and dependent variables with the chosen responses and assigned goals of MTC–loaded lipidic nano-vesicular hybrids.

Independent variables	Low	Medium	High	Units
X_1_: Chitosan amount	0	25	50	mg
X_2_: Cholesterol conc.	0.5	0.75	1	Molar ratio
X_3_: PEG 600 conc.	0	15	30	%
X_4_: Tween 80 conc.	0	15	30	%
Dependent variables	Units	Goals		
Y_1_: Mean vesicle size	nm	Minimize		
Y_2_: Zeta potential	mV	Maximize		
Y_3_: Entrapment efficiency	%	Maximize		
Y_4_: Initial release	%	Maximize/ Minimize		
Y_5_: Cumulative release	%	Maximize/ Minimize		

**Table 2.** Experimental runs and observed values of responses for DLD MTC–loaded lipidic nano-vesicular hybrids, along with the two optimized formulations (Opti-Max and Opti-Min MTC–loaded PEG-T-Chito-Lip nano-vesicular formulations).^a^

Runs (#)	Factors				Responses									
	X_1_	X_2_	X_3_	X_4_	Y_1_		Y_2_		Y_3_		Y_4_		Y_5_	
	*(mg)*	*(Molar ratio)*	*(%)*	*(%)*	*(nm)*	± SD	*(mV)*	± SD	*(%)*	± SD	*(%)*	± SD	*(%)*	± SD
F1	0	0.5	0	0	212.60	2.60	−27.3	0.65	59.60	0.86	27.3	3.6	95.7	4.3
F2	50	0.5	0	30	392.84	1.33	41.2	0.89	91.33	2.73	18.8	0.9	81.5	0.6
F3	50	1	0	0	456.67	2.69	45.9	0.50	89.45	1.38	3.4	0.7	68.8	0.9
F4	25	0.75	15	0	357.23	1.51	41.5	0.67	79.36	1.59	9.4	0.8	71.9	3.8
F5	50	1	30	0	517.33	3.96	37.8	0.68	89.95	1.60	2.6	0.4	51.9	1.6
F6	25	1	15	15	378.27	0.98	40.4	0.95	84.67	0.80	5.3	0.6	74.4	4.1
F7	25	0.75	30	15	455.43	3.50	35.6	0.98	86.21	2.80	10.2	1.5	82.7	2.7
F8	25	0.75	0	15	336.10	1.60	47.8	1.05	77.93	0.33	15.9	0.7	89.5	2.4
F9	25	0.5	15	15	340.95	0.85	46.7	0.97	82.58	2.54	19.4	0.5	91.7	0.8
F10	50	0.75	15	15	437.80	0.57	39.9	0.45	90.39	3.56	9.3	1.6	75.6	1.7
F11	0	1	30	30	387.31	2.83	−35.9	0.63	74.15	1.73	12.3	0.8	83.1	2.2
F12	50	0.5	30	30	451.46	1.33	30.1	0.88	93.85	3.35	8.9	0.2	72.4	2.9
F13	0	0.5	30	0	297.17	2.64	−32.4	0.64	66.30	1.46	16.5	1.2	87.6	1.7
F14	0	1	0	30	321.36	1.42	−29.5	0.95	71.30	2.24	10.8	1.9	82.9	3.8
F15	25	0.75	15	30	377.40	2.08	38.2	1.67	85.13	1.80	15.4	1.1	87.3	3.4
F16	0	0.75	15	15	283.20	0.64	−34.6	0.94	69.18	3.60	16.6	1.7	89.1	4.1
F17	25	0.75	15	15	326.53	1.06	40.3	0.49	85.35	4.30	12.2	0.9	86.4	2.4
F18	25	0.75	15	15	304.68	1.82	41.4	0.86	88.42	2.25	17.9	0.8	88.7	3.8
F19	25	0.75	15	15	349.30	0.95	38.9	0.48	82.68	2.90	15.6	0.5	91.8	2.7
Opti-Max	26.5	0.5	0.9	22	344.90	1.84	39.7	2.33	79.64	0.89	21.9	2.7	95.6	3.9
Opti-Min	31.8	1	10.7	0.05	405.67	3.67	34.8	3.15	86.45	2.91	5.3	0.6	64.2	4.3

^a^ All formulations were formulated with 1 molar ratio Span 60. X_1_ is the chitosan amount (mg), X_2_ is the cholesterol molar ratio concentration, X_3_ is the PEG 600 concentration percentage, X_4_ is the Tween 80 concentration percentage, Y_1_ is the mean vesicle size (nm), Y_2_ is the zeta potential (mV), Y_3_ is the entrapment efficiency percentage, Y_4_ is the percentage of MTC initial release after 1 h and Y_5_ is the percentage of MTC cumulative release after 24 h.

### 2.3. Formulation of DLD MTC–loaded lipidic-nano-vesicular hybrids

The blank (MTC-free) lipidic nanovesicles, chitosan-lipidic nanovesicles, PEGylated chitosan-lipidic nanovesicles, Tween 80–functionalized chitosan-lipidic nanovesicles, and PEGylated Tween 80–functionalized chitosan–lipidic (PEG-T-Chito-Lip) nanovesicles (in addition to the MTC–loaded nano-vesicular preparations) were created according to the composition of each formulation listed in Table 2, using a modified thin-film hydration technique (Chen et al., 2019).

For the elaboration of MTC-free and MTC–loaded lipidic nanovesicles, mixtures of Span 60 and cholesterol (according to the specified amounts listed in Table 2, with final weight of 100 mg) were dissolved in organic solvent (chloroform), and the solvent was then evaporated under pressure (at 58-60 °C) to form a thin film on the inner walls of the rounded-bottom container fixed to a Büchi-M/HB-140 rotary evaporator (Flawil, St. Gallen, Switzerland). After that, a hydration medium (10 mL phosphate buffer saline; PBS) was added, allowing for a brief period of time at a temperature above the lipid phase transition temperature (58-60 °C) for the film to swell. Unilamellar vesicles are created by further processing vesicles that are formed when the container is slightly shaken/rotated with the aid of 7–10 glass beads (Chen et al., 2019). Chitosan, PEG 600, and/or Tween 80 were used with the specified amounts listed in Table 2 to create the different nano-vesicular hybrids, along with the 100 mg mixture of Span 60 and cholesterol.

To create the chitosan-lipidic nanovesicles, transparent chitosan solution with a 0.5% w/v concentration was created by dissolving 1 gm of chitosan in 200 ml of aqueous 3% acetic acid. This solution was then agitated using a magnetic stirrer at 25 °C for 24 h before being meticulously filtered. Then, 10 mL of the filtered chitosan solution was added to the previously prepared lipid mixtures’ film (with using enough acetic acid to reach a pH of 3.5-4.5) and used as a hydration medium. In the film synthesis process, PEG 600 or Tween 80 was added to the 100 mg total lipid mix in order to create PEGylated or Tween 80–functionalized-lipidic nanovesicles, respectively (Megahed et al., 2022). For drug-loaded nanovesicles, 50 mg of MTC was dissolved in the 10 mL hydration medium and used accordingly. The same technique was used to create blank nanovesicles without adding MTC to the formulations. After preparation of all formulations, they were kept in freezer at −20 °C for further investigation (Arafa & Ayoub, 2017).

### 2.4. Characterization of the DLD MTC-loaded lipidic-nano-vesicular hybrids

#### 2.4.1. Determination of vesicle size and zeta potential

Using dynamic light scattering (DLS), which is based on laser diffraction at room temperature (NICOMP^TM^ 380 ZLS NICOMP particle sizing system, Santa Barbara, California, USA), the vesicle size distribution by intensity and zeta potential for each formula was measured (Abdulkarim et al., 2015; Li et al., 2016; Marianecci et al., 2012, 2010; Wu et al., 2016). Each sample was assessed three times in total. As an additional indicator of homogeneity, the polydispersity index (PDI) was also measured.

#### 2.4.2. Determination of entrapment efficiency (EE%)

The frozen dispersions of DLD MTC–loaded nano-vesicular hybrids were thawed above the lipid phase transition temperature (above elaboration temperature; > 58-60 °C). According to a prior study, freeze-thawing significantly increased the entrapment efficiency (Mokhtar et al., 2008). By cooling centrifugation at 10,000 rpm and at −4 °C temperature with the large capacity table-top refrigerated centrifuge (Centurion Scientific Ltd., Stoughton, UK), the free drug in the supernatant was separated from the entrapped MTC (Li et al., 2014). Then, the nano-vesicular pellets were twice-washed with 30 mL of PBS pH 7.4, and the volume of the collected supernatant was set to 100 mL. By measuring the un-entrapped drug in the washing and deducting it from the total initial amount of drug used at the start of the MTC–loaded nano-vesicular hybrid preparations using a spectrophotometric assay (Jasco V-630, Tokyo, Japan) at 269 nm wavelength, it was possible to estimate the amount of drug entrapped (EE%). Each experiment was carried out three times, and the drug-free nano-vesicular hybrid dispersions were treated equally and taken as a control.

#### 2.4.3. Determination of the initial and cumulative release percentage

To simulate the *in-vivo* behavior of nano-vesicular hybrids and to determine the initial release percentage (at 1 h) and cumulative release percentage (at 24 h) of MTC for the completion of the design responses observation and evaluation, the *in-vitro* release study for each MTC–loaded nano-vesicular formulation of the DLD was carried out (Bayindir & Yuksel, 2010). The study was done in triplicate. The amount of MTC encapsulated was determined after each preparation was separated and thoroughly double-washed. The total amount of drug was considered as the amount retained at zero time. The pellet of each preparation was then accurately diluted to 10 mL in PBS with a pH 7.4. The experiment was conducted using a rotary shaker (GLF 3203; Hilab, Düsseldorf, Germany). The device was set to 150 stroke/min, and the temperature was set to 37 °C plus or minus 0.5 °C (Danhier et al., 2009). One mL of the nano-vesicular suspension was sampled at the following intervals: 1, 2, 3, 4, 5, 6, 8, 12 and 24 h. The samples were separated and washed. Then, the collected supernatant was used for measurement using the aforementioned spectrophotometric assay (section 2.4.2.). Consequently, the amounts of MTC released and retained for each formulation were computed for each time interval (Jana et al., 2014).

To learn more about the release mechanism, one can theoretically model the MTC release characteristics from nano-vesicular hybrids. The collected release data were fitted to a variety of kinetic models, including the Higuchi and Korsmeyer-Peppas models, which are frequently used to describe how drugs are released from different types of nanovesicles (El-Say et al., 2015; Jana et al., 2014; Megahed et al., 2022).

### 2.5. Prediction and characterization of Opti-Max/Opti-Min MTC–loaded PEG-T-Chito-Lip nano-vesicular formulations

Analysis of variance and multiple response optimization were used to predict the optimized MTC–loaded PEG-T-Chito-Lip nanovesicular hybrids using the statistical tool Statgraphics^®^ Centurion 18 Software (StatPoint, Inc., Warrenton, VA, USA). Prediction was performed twice according to the specified goals, one for the maximized release goal; assigned as Opti-Max MTC–loaded PEG-T-Chito-Lip nano-vesicular formulation, and the other for minimized release goal; assigned as Opti-Min MTC–loaded PEG-T-Chito-Lip nano-vesicular formulation. The measurements included the mean vesicle size, zeta potential, entrapment efficiency, the percentage of MTC initial release after 1 h, and the percentage of MTC cumulative release after 24 h, which were performed for the two elaborated optimized formulations. These responses were the same as those measured for the previously DLD formulations. This was carried out to contrast the actual responses with those that the DLD results had predicted for both Opti-Max and Opti-Min formulations. Three replicas of each response measurement were made. Further research and characterization were also conducted for Opti-Max and Opti-Min formulations including light and electron microscopical inspection, differential scanning calorimetry and thermogravimetric analysis. Subsequently, the two optimized formulations were combined to make the dual-optimized MTC–loaded PEG-T-Chito-Lip nano-vesicular formulation, which was loaded into an *in-situ* gel base to be furtherly scaled up and undergone *in-vivo* pharmacokinetics/nose-to-brain delivery evaluation on Sprague-Dawley rats.

#### 2.5.1. Light microscopy

Light microscopy was used to analyze the morphological features of the individual optimized formulations (Opti-Max and Opti-Min). The optimized formulations were studied using an optical microscope (Leica DM300, Wetzlar, Germany) equipped with a 40× lens and a 16× eyepiece, and then it was shot using a digital camera (iPhone 12, Apple, USA) (Abaee & Madadlou, 2016; Abdelbary & El-gendy, 2008; Yuksel et al., 2016).

#### 2.5.2. Transmission electron microscopy (TEM)

The optimized formulations (Opti-Max and Opti-Min MTC–loaded formulations) were individually imaged using TEM (Zeiss EM 10, Oberkochen, Germany) after the sample had been negatively stained. The potassium phosphotungstate was sufficiently dissolved in distilled water to form a 1% (w/v) dye, and then agitated to give a transparent solution. One drop of the formulation under test and one drop of the newly created dye were combined, which was then allowed to dry before being examined by the TEM (Abdelmonem et al., 2021; El-Ridy et al., 2011; ELhabal et al., 2022; Kassem et al., 2017).

#### 2.5.3. Thermogravimetric analysis (TGA) and Differential Scanning Calorimetry (DSC)

DSC and TGA were employed to examine how medicines interact with the compositions of the formulated lipidic-based nanovesicles (Abdelkader et al., 2011; Mulik et al., 2012).

The dehydrated pellets of the two distinct optimized formulations (Opti-Max and Opti-Min) and the raw/pure MTC were subjected to TGA and DSC. Each formulation was produced, separated, twice-washed, and dehydrated using a freeze drier. On the Shimadzu DSC-50 Differential Scanning Calorimeter (Shimadzu Corporation, Kyoto, Japan), all thermograms were recorded. Over a temperature range of 20–400 °C, a heating rate of 5 °C/min was used, along with nitrogen purging at a rate of 100 mL/min (Desoqi et al., 2021; El-Nabarawi et al., 2013; Gardouh et al., 2021; Gurrapu et al., 2012; Nasr et al., 2008). The DSC thermograms’ computer reports were given and evaluated.

### 2.6. Assimilation of thermosensitive in-situ gel (ISG) with the dual-optimized MTC–loaded PEG-T-Chito-Lip nano-vesicular formulation for intranasal application

The Opti-Max and Opti-Min MTC–loaded PEG-T-Chito-Lip nano-vesicular formulations were combined to make the dual-optimized MTC nano-vesicular formulation (with both maximized and minimized release goals). Poloxamer 407 (22% w/v) and Carbopol 940 (0.5% w/v) were combined by cold technique to create a suitable *in-situ* gel (ISG) for the intranasal application of the dual-optimized MTC–loaded PEG-T-Chito-Lip nano-vesicular formulation with an MTC level of 0.5% w/v. Poloxamer and Carbopol concentrations were chosen based on the polymeric concentrations that result in satisfactory ISG formulation characterization and are in good accord with a previously published work (Ahmed et al., 2019). In a nutshell, the dual-optimized MTC–loaded PEG-T-Chito-Lip nano-vesicular formulation was aqueously cold dispersed at 4 °C on a magnetic stirrer, and the predicted amounts of poloxamer and Carbopol were added successively. The resulting dispersions were stored overnight at 6 °C. The same procedure used above was also used to prepare ISG formulation loaded with raw MTC (0.5% w/v).

### 2.7. *Valuation of nose-to-brain delivery and* in-vivo *pharmacokinetics of the dual-optimized MTC–loaded PEG-T-Chito-Lip nano-vesicular ISG formulation*

#### 2.7.1. Conditions for chromatography

Any samples (plasma or organ homogenate) that included MTC were identified in this study utilizing an isocratic HPLC chromatographic procedure with modification (Zaki et al., 2007, 2006b). Along with a quaternary pump, autosampler, vacuum degasser, and Winchrom software, the HPLC equipment (Parkin Elmer, Boston, MA, USA) was set up with an adjustable wavelength ultraviolet-spectroscopic detector set at 269 nm. On a Waters C18 3.9300 mm Bondapak column (Waters, Milford, MA), the chromatographic separation was carried out at ambient temperature. The mobile phase was pumped at a flow rate of 1.5 mL/min and had an acetonitrile: 20 mM potassium dihydrogen phosphate buffer solution (pH 3.5, corrected with orthophosphoric acid) ratio of 44:56 v/v.

For MTC extraction from the plasma samples, 0.25 mL of acetonitrile was added to a 0.25 mL aliquot of the sample, which was then subjected to 1 min of vigorous vortex shaking and 10 min of 5000 rpm centrifugation. The organic solvent was separated by evaporation at 50 °C under a nitrogen stream until it was completely dry. A 20 µL injection volume was added after the residue was reconstituted in 80 µL of mobile phase. By combining a predetermined quantity of paracetamol (as internal standard; IS) with a known volume of the mobile phase to create a 100 ng/mL solution, an IS solution was created.

The chromatographic conditions used in this study were tested internally and were deemed to be sensitive, exact, reliable, accurate, and selective. With a regression coefficient (R^2^) of 0.9998, the limits of MTC quantitation and detection were 25 and 2000 ng/mL, respectively. MTC and IS had retention times of 3.9 and 6.9 min, respectively. At 25 ng/mL (lower limit of quantification; LLOQ) and 2000 ng/mL (upper limit of quantification; ULOQ), the mean MTC recovery was 107.1% and 100.2%, respectively.

#### 2.7.2. Procedure and handling of animals

In this investigation, 84 male Sprague Dawley rats (165-215 g body weight) were used (twenty-one rats per group). This quantity of rats was deemed sufficient for blood/organ sample collection using an alternative technique. A single-dose, one-period parallel design was employed in this investigation. The animal study protocol was approved by the Cairo University Faculty of Pharmacy’s Research Ethics Committee (Serial no: PI-2763). The study was carried out in accordance with the International Conference on Harmonization (ICH), European Medicines Agency (EMA), Good Clinical Practice (GCP), and Food and Drug Administration (FDA) regulations. The research was conducted in accordance with the Declaration of Helsinki, the Guiding Principles in the Care and Use of Animals (DHEW production NIH 8023), and the ‘Standards of Laboratory Animal Care’ (NIH distribution #8523, revised in 1985). The rats were housed in cages with 12-h light/dark cycles at a temperature of 25 °C and a relative humidity of 55 ± 10%. The rats were kept with unlimited access to water. Environmental and general conditions were closely watched.

**Figure 1.**
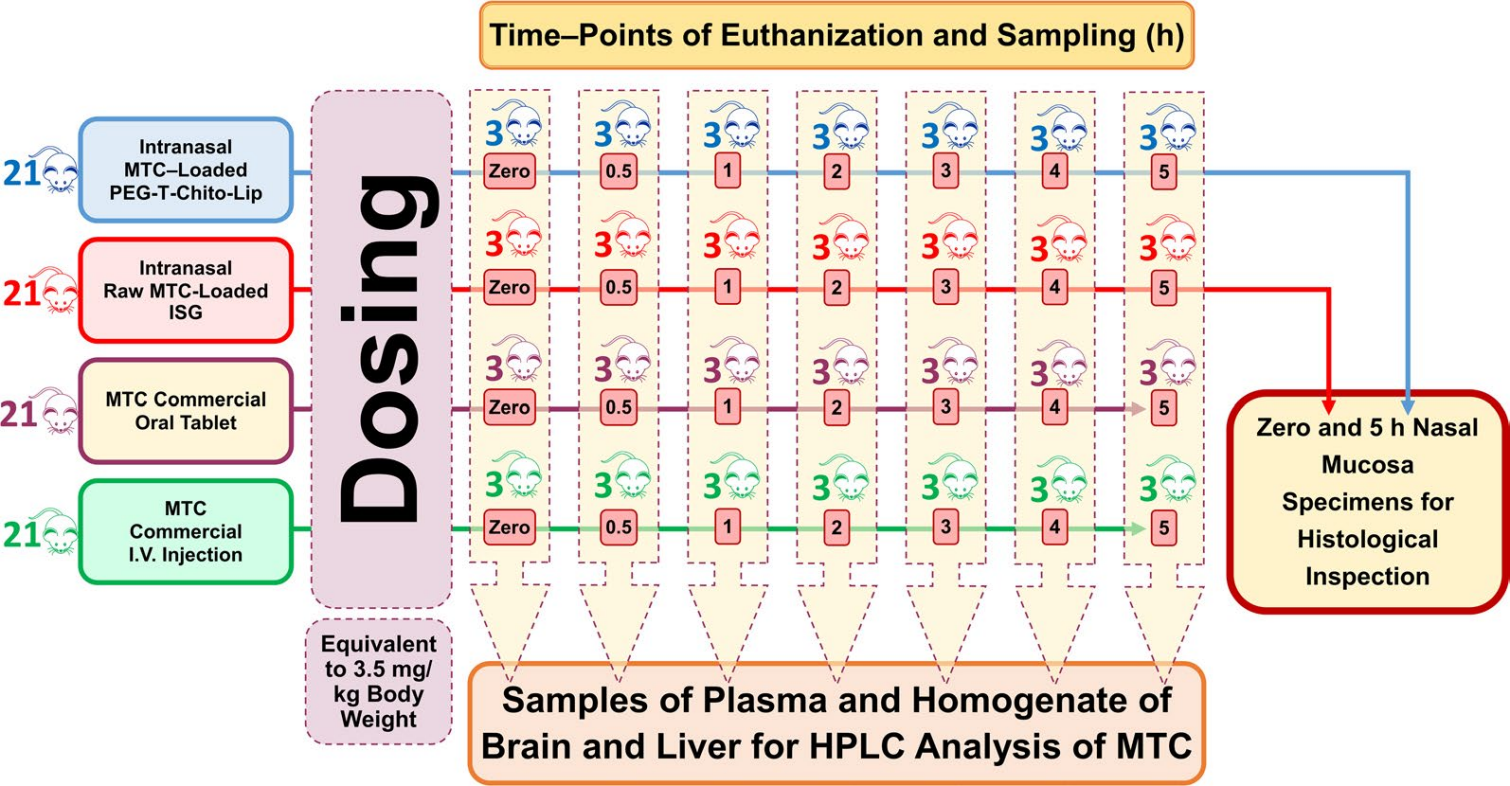
Schematic illustration of the performed valuation of nose-to-brain delivery and *in-vivo* pharmacokinetics of the intranasal dual-optimized MTC–loaded PEG-T-Chito-Lip nano-vesicular ISG. Abbreviations: MTC, metoclopramide hydrochloride; PEG-T-Chito-Lip nano-vesicular hybrid, PEGylated Tween 80–functionalized chitosan–lipidic nano-vesicular hybrid; ISG, *in-situ* gel; I.V., intravenous.

The eighty-four rats were divided into 4 groups of 21 rats. A dose corresponding to 3.5 mg/kg body weight was given to two groups (test groups) *via* intranasal administration of two different drug formulations: one received the dual-optimized MTC–loaded PEG-T-Chito-Lip nano-vesicular ISG and the other received the raw MTC-loaded ISG. The third and fourth (reference) groups were administered the MTC commercial oral tablet (Primperan^®^ 10 mg) and MTC injection (Primperan^®^, 10 mg per 2 mL), respectively, with a dose equivalent to 3.5 mg/kg body weight (Pero et al., 1997). Through esophageal intubation, the oral-reference group was given 1 mL of 0.5% carboxymethyl cellulose aqueous solutions that contained the pulverized MTC tablet (equal to 3.5 mg/kg body weight). A 3.5 mg/kg body weight intravenous (I.V.) bolus injection of MTC was given to the injection-reference group in the left lateral tail vein. In Figure 1, the methods used for the *in-vivo* experiments are schematically depicted.

#### 2.7.3. Criteria of MTC dose selection

MTC dosing generally varies between 10 and 20 mg three to four times daily. Regarding chemotherapy-associated nausea and vomiting, MTC dosing regimen can be as high as 2 mg per kg body weight (or higher) intravenously (I.V.) five times, with concomitant diphenhydramine or lorazepam to decrease extrapyramidal reactions (EPRs) incidences with MTC high doses (Allen et al., 1985; Herrstedt et al., 2022). Depending on literature and previous research works on MTC, the dose amount was chosen as the human MTC single dose widely ranged from 0.17 to 2–3 mg/kg, with equivalent rat single dose range from 1–12.5 mg/kg body weight, according to the following equation (Reagan-Shaw et al., 2008):

(1) $$\text{Human equivalent dose}(\mathrm{mg}/\mathrm{kg})=\text{animal dose}(\mathrm{mg}/\mathrm{kg})\;\times \;\frac{\text{rats Km }(6\;\mathrm{kg}/m^{2})}{\text{human Km }(37\;\mathrm{kg}/m^{2})}$$

where: Km = Body weight (kg)/Body surface area (m^2^). Accordingly, the dose selection was done in order to be in the low-mid region of this wide range of doses (between 1 and 4 mg/kg) to avoid any undesirable side effects during the study, and also to be high enough for quantification. Therefore, the 3.5 mg/kg MTC dose for rats can be considered suitable enough for this *in-vivo* comparative study.

#### 2.7.4. Sampling procedure

In each group, the twenty-one rats were divided into 7 subgroups (3 rats per subgroup). Each subgroup was assigned to a time interval for both blood and organ sampling. The rats were euthanized immediately after blood sampling, and afterward, brain and liver tissues were collected.

At 0, 0.5, 1, 2, 3, 4, and 5 h following drug administration, blood samples (250 µL) from all groups were obtained through retro-orbital punctures performed under light ether anesthesia. Samples collected at zero time and just before I.V. injection administration were assigned as blank samples (normal-control). The plasma from the collected blood samples was separated by centrifuging at 3000 rpm for 5 min, and the plasma was then permitted to be stored at −80 °C. For the homogenate samples, liver and brain tissue samples that were precisely weighed at 1 gm each were immediately rinsed with normal saline and subjected to tissue homogenization using a probe tissue homogenizer at a pace of 4000 rpm for 3 min in 5 mL of 50 mM Tris-HCl buffer (Megahed et al., 2022). The acquired tissue homogenate samples were then placed in freezers and kept at −80 °C until the determination of MTC concentration using the aforementioned HPLC method.

#### 2.7.5. Valuation of pharmacokinetic parameters

The pharmacokinetic characteristics of the intranasal dual-optimized MTC–loaded PEG-T-Chito-Lip nano-vesicular ISG were assessed versus those of the intranasal raw MTC-loaded ISG, the MTC commercial oral tablet, and the MTC commercial I.V. injection. MTC concentrations in the plasma, brain, and liver were measured and used in estimating/calculating pharmacokinetic (PK) parameters.

The non-compartmental extravascular PK model was used to compute and specify the following PK parameters using an add-in for Excel (PKsolver). The peak/highest plasma concentration (C_max_) and its time (T_max_) were measured. Areas under the curve (AUC_0–t_ and AUC_0–∞_) and further PK parameters as the half-life (t_1/2_), the elimination rate constant (K_el_), volume of distribution (V_d_), the mean residence time (MRT_0–∞_), and the apparent total body clearance (Cl) were also considered. The absolute and relative bioavailability values of the dual-optimized MTC–loaded PEG-T-Chito-Lip nano-vesicular ISG (relative to raw MTC-loaded ISG and the reference groups) were determined (Abo-EL-Sooud, 2018).

#### 2.7.6. Calculation and evaluation of the nose-to-brain delivery parameters of the dual-optimized MTC–loaded PEG-T-Chito-Lip nano-vesicular ISG formulation

For each formulation, a brain-to-blood ratio was determined and examined. Besides, the ability of the intranasal formulations to target the brain through the nasal route has been assessed using drug targeting efficiency (DTE) and direct transport percentage from the nose to the brain (DTP), where DTE denotes the average time of partitioning ratio of MTC among the brain and the blood, while DTP signifies the percentage of MTC delivered to the brain *via* the direct-transport route (Sayyed et al., 2022). The uptake from the nose to the brain following nasal delivery can be determined using the DTP of the drug to the brain which calculated by dividing the AUC for the brain and blood (Equation (2)). The DTE and DTP calculations were performed using the following formulae/equations.

(2) $$\mathrm{DTE}\%=\frac{{\mathrm{AUC}}_{\mathrm{Brain}-\mathrm{Intranasal}}}{{\mathrm{AUC}}_{\mathrm{Plasma}-\mathrm{Intranasal}}}\times 100$$

The AUC used here is that of nasal delivery of MTC for both the blood and brain from time zero to 5 h after intranasal administration, where AUC*_Brain-Intranasal_* and AUC*_Plasma-Intranasal_* indicate that for the brain and the plasma, respectively. For estimating the DTP, a prior calculation of the AUC of the brain fraction (AUC*_bf_* produced from the blood systemic circulation of the formulation after intranasal administration) should be done from the following equation.

(3) $${\mathrm{AUC}}_{bf}=\frac{{\mathrm{AUC}\;}_{\mathrm{Brain}-I.V.}}{{\mathrm{AUC}\;}_{\mathrm{Plasma}-I.V.}}\times {\mathrm{AUC}}_{\mathrm{Plasma}-\mathrm{Intranasal}}$$

where AUC*_Brain–I.V._*is the AUC_0–t_ for the brain after I.V. injection of MTC, AUC*_Plasma–I.V._* is the AUC_0–t_ for the plasma after I.V. injection of MTC, and AUC*_Plasma–Intranasal_* is the AUC_0–t_ of the plasma following intranasal administration of the dual-optimized MTC–loaded PEG-T-Chito-Lip nano-vesicular ISG (as well as the raw MTC-loaded ISG intranasal for comparison with the dual-optimized formula).

After the aforementioned calculation of AUC*_bf_*, the DTP can now be estimated using the following formula:

(4) $$\mathrm{DTP}\%=\frac{{\mathrm{AUC}}_{\mathrm{Brain}-\mathrm{Intranasal}}-{\mathrm{AUC}}_{bf}}{{\mathrm{AUC}}_{\mathrm{Brain}-\mathrm{Intranasal}}}\times 100$$

In this equation, AUC*_Brain-Intranasal_* is the AUC_0–t_ for the brain after intranasal application.

By comparing the AUC for brain and blood for both intranasal administrations relative to MTC intravenous injection, the drug targeting index (DTI) was computed for both the dual-optimized MTC–loaded PEG-T-Chito-Lip nano-vesicular ISG and the raw MTC-loaded ISG:

(5) $$\mathrm{DTI}=\frac{{\mathrm{AUC}}_{\mathrm{Brain}-\mathrm{Intranasal}}/{\mathrm{AUC}}_{\mathrm{Plasma}-\mathrm{Intranasal}}}{{\mathrm{AUC}\;}_{\mathrm{Brain}-I.V.}/{\mathrm{AUC}\;}_{\mathrm{Plasma}-I.V.}}$$

It is worthy to mention that all brain targeting (nose-to-brain delivery) parameters were assessed for both the dual-optimized MTC–loaded PEG-T-Chito-Lip nano-vesicular ISG and the raw MTC-loaded ISG.

#### 2.7.7. Histological examination of nasal mucosa

Microscopic examination of the nasal epithelium of treated rats was carried out to investigate any alteration or irritation in the intranasal tissues that could result from the intranasal application of the dual-optimized MTC–loaded PEG-T-Chito-Lip nano-vesicular ISG and the raw MTC-loaded ISG.

In each test group, the subgroup of five-hour-euthanized rats had their nasal mucosa meticulously removed from the nasal cavity. All samples of nasal mucosa were taken out, preserved in 10% formalin, dried up, and then embedded in paraffin wax. Samples were examined using a Nikon Eclipse 80i digital imaging light microscope (Kanagawa, Japan) after being cut into 4-micron sections and stained with hematoxylin and eosin (H&E). The control sample was also examined for comparative investigation.

The normal/control samples were taken from subgroups at time zero (before to the administration of the ISG formulations). The appearance of epithelial and goblet cells, as well as any indication of irritation/inflammation, were all examined in each tissue sample (Ahmed et al., 2019; Hamza et al., 2022).

#### 2.7.8. Statistical assessment

All collected data were statistically analyzed using GraphPad Prism, version 8.4.2 Software (San Diego, CA, USA). Two-way ANOVA followed by Tukey’s multiple comparisons test was used to compare each mean with the others at all time points and determine the significance between groups in relation to the plasma, brain, and liver homogenate concentration-time curves. Two-tailed unpaired t-tests were performed on the formulation’s pharmacokinetic characteristics to determine their significance in relation to one another. Results were deemed significant if *P* < 0.05.

## 3. Results and discussion

By using the selected factors X_1_, X_2_, X_3_, and X_4_ at various levels, as shown in Table 2, 19 nano-vesicular formulations were created. Chitosan concentrations at three different levels were employed as X_1_ (0, 25 and 50 mg). Three distinct ratios of the cholesterol levels (molar ratio) were employed as X_2_ (0.5, 0.75 and 1). Last but not least, three different concentrations of PEG 600 and Tween 80 were applied as X_3_ and X_4_, respectively (0, 15 and 30 percent). It is noteworthy to note that in all formulations, the surfactant concentration (Span 60) was set to be constant (1 molar ratio concentration). The data for vesicle size, zeta potential, entrapment efficiency, initial and cumulative percentage of drug release were significantly varied with the various abovementioned factors’ levels (Table 2). The statistical programme Statgraphics^®^ Centurion 18 Software (StatPoint, Inc., Warrenton, VA, USA) was used to analyze the polynomial equations that represented the mathematical relationships between the factors and the observed responses for their significance. The results of this analysis were evaluated and interpreted in the following sections.

It is also crucial to note that the zeta potential results (Y_2_) for any formulation with a negative value of zeta potential were applied as numerical values without considering the negative sign of the charge. For maximizing the zeta potential of the generated nanovesicles and preventing any data misuse by the software, the aforementioned action is done in order to correctly represent the mathematical correlations between the components and the observed response Y_2_. Nevertheless, Table 2 showed the actual zeta potential values (together with the corresponding charge).

### 3.1. Characterization of the DLD MTC-loaded lipidic-nano-vesicular hybrids and assessment of responses

#### 3.1.1. Vesicle size and zeta potential

Vesicles size, PDI, and zeta potential are significant factors in determining how stable and effective nanovesicles are and how they affect the biopharmaceutical properties of nanovesicles as nano-carriers (Marianecci et al., 2010). Due to this, the vesicular size and zeta potential of nanovesicles were assessed and assigned as formulation parameters of DLD.

From Table 2, the formulated nanovesicles had sizes ranging from 212.6 nm for F1 to 517.33 nm for F5. In agreement with previously published data, MTC nano-vesicular formulations showed low PDI ranging from 0.341 to 0.662, indicating a relatively accepted particle size distribution. (El-Say et al., 2016; Marianecci et al., 2014, 2012; Sahoo et al., 2014). Additionally, the DLD formulations’ zeta potentials ranged from −27.3 mV (as a numerical value) for F1 to 47.8 mV for F8 (also as a numerical value), showing that all DLD formulations have good stability, which is consistent with many other research works (Kumar et al., 2015; Marianecci et al., 2014; Sahoo et al., 2014).

The formulation F1 without chitosan, PEG 600, or Tween 80 produced the smallest vesicles possible using Span 60 at a 1 molar ratio and cholesterol at a 0.5 molar ratio. The highest vesicle size was found in F5, which also contained 50 mg of chitosan and 30% PEG 600, along with Span 60 and cholesterol at a 1:1 molar ratio concentration. As a result, increasing cholesterol concentration caused an increase in mean vesicle diameters, which is consistent with earlier research (Bendas et al., 2013). Moreover, the addition of chitosan and PEG 600 was found to have significant influence on vesicle size (Hassanzadeganroudsari et al., 2019; Kaur & Mehta, 2017; Mahmoud et al., 2017; Megahed et al., 2022; Waddad et al., 2013).

#### 3.1.2. Entrapment efficiency (EE%)

The observed values of the entrapment efficiency for DLD formulations were displayed in Table 2. The prepared nanovesicles’ entrapment efficiency was found to range from 59.6 to 93.85%. The results showed that increasing the amounts of chitosan, PEG 600, and Tween 80 used to formulate nanovesicles increases the efficiency with which MTC is trapped in the nano-vesicular hybrids. This was in accordance to many previous studies (Hassanzadeganroudsari et al., 2019; Kaur & Mehta, 2017; Mahmoud et al., 2017; Megahed et al., 2022; Waddad et al., 2013). Besides, the amount of chitosan was found to be the most significant effective change that influence EE%. Additionally, when the amount of cholesterol raised, the percentage of entrapment efficiency increased as well. The creation of less leaky vesicles and an increase in EE% will follow an increase in membrane stiffness brought on by an increase in cholesterol levels. Cholesterol will therefore reduce the fluidity of nano-vesicular hybrids and give vesicles a stiffness. It delays the change from the gel to the liquid phase, which results in less leaky vesicles. (Abdelbary & El-gendy, 2008; Sahoo et al., 2014; Waddad et al., 2013).

#### 3.1.3. In-vitro release profiles and kinetic treatment of the release data

**Figure 2.**
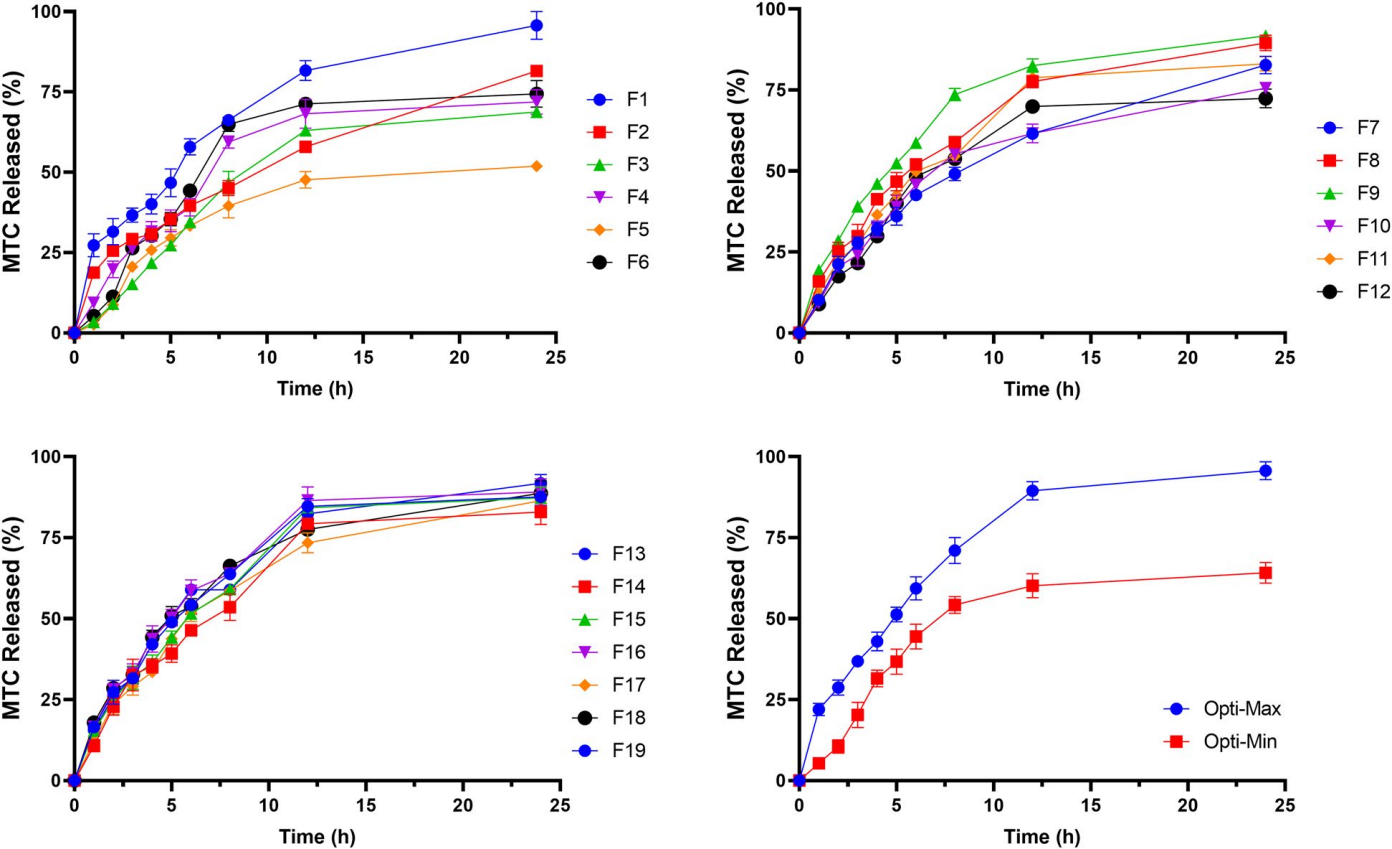
*In-vitro* release profile of MTC from different DLD formulations, Opti-Max and Opti-Min MTC–loaded PEG-T-Chito-Lip nano-vesicular formulations. Abbreviations: MTC, metoclopramide hydrochloride; PEG-T-Chito-Lip nano-vesicular hybrid, PEGylated Tween 80–functionalized chitosan–lipidic nano-vesicular hybrid.

Table 2 and Figure 2 provide the *in-vitro* release profiles of DLD formulations as well as the percentage of MTC released after 1 h to indicate initial release and after 24 h to indicate cumulative release. The findings showed that F1 had the highest initial MTC release rate (27.3%), while F5 had the lowest initial MTC release percentage (2.6%). Additionally, F1 released the highest percentage of cumulative MTC (95.7%) while F5 released the lowest percentage of cumulative MTC (51.9%).

The formulation’s utilization of higher cholesterol concentrations, which significantly reduced the drug’s efflux and is consistent with its capacity to stabilize membranes, provided the most reliable explanation for these results (El-Nabarawi et al., 2013; Guinedi et al., 2005). Although the effect of chitosan amount as well as PEG 600 and Tween 80 concentrations were also found significant, the most effective factor was the cholesterol concentration (Bendas et al., 2013). The quick-release phase of the release profile may be explained by the drug’s desorption from the surface of nanovesicles, while the plateau shape of the release profile may be primarily caused by the drug’s diffusion from the formed vesicles (Megahed et al., 2022; Pardakhty et al., 2007).

The outcomes of the mathematical modeling of the prepared nanovesicles’ release mechanism were shown in Table 3. According to the results, MTC was released from DLD nano-vesicular formulations F9, F10, F17, F18, and F19 in a second-order manner, while MTC was released from formulations F1, F2, F7, and F8 in a first-order manner (Abdelkader et al., 2011; Gardouh et al., 2021; Kumar & Rajeshwarrao, 2011; Ruckmani & Sankar, 2010). Additionally, the Higuchi-diffusion kinetics pattern was followed for the release of MTC in the remaining DLD formulations (Desoqi et al., 2021; Megahed et al., 2022). Moreover, kinetic treatment showed that the (n) values for the same formulations were established a non-Fickian diffusion pattern for the release of MTC from almost all DLD nano-vesicular formulations, confirming that the release is governed *via* a combination of polymer relaxation/erosion along with diffusion mechanism (Kassem et al., 2017; Mehta & Jindal, 2013).

**Table 3.** Kinetics studies of the release profiles of MTC from different DLD formulations, as well as the two optimized formulations (Opti-Max and Opti-Min MTC–loaded PEG-T-Chito-Lip nano-vesicular formulations).

Formulations	(r) value				Korsmeyer-Peppas model (n) value^a^	Release Rate constant
	Zero order	First order	Second order	Higuchi Diffusion model		
F1	0.935321	**–0.99731**	0.949483	0.978033	0.44475137	−0.127630
F2	0.986926	**–0.99818**	0.973069	0.996403	0.46061692	−0.063720
F3	0.898888	−0.93767	0.961799	**0.961944**	0.99187883	18.90613
F4	0.866456	−0.90640	0.934575	**0.942258**	0.66274254	17.47046
F5	0.838950	−0.88444	0.921519	**0.933285**	0.92237558	13.11036
F6	0.836004	−0.88437	0.918715	**0.924240**	0.88219142	19.85236
F7	0.955236	**–0.99835**	0.983468	0.995524	0.63954735	−0.069830
F8	0.917845	**–0.98835**	0.987232	0.977366	0.57221493	−0.09258
F9	0.876106	−0.97415	**0.993392**	0.955028	0.52374740	0.004853
F10	0.899956	−0.96526	**0.996377**	0.968950	0.66510038	0.001315
F11	0.893591	−0.94247	0.959881	**0.960843**	0.63278352	19.72367
F12	0.857584	−0.90648	0.935262	**0.940096**	0.70925395	18.09740
F13	0.879566	−0.93564	0.952126	**0.953743**	0.56301198	19.94656
F14	0.897427	−0.93762	0.949219	**0.960782**	0.64463406	19.61249
F15	0.894940	−0.93751	0.948753	**0.959247**	0.58513901	20.34569
F16	0.873153	−0.93411	0.949304	**0.950573**	0.56227996	20.16422
F17	0.912531	−0.9848	0.994240	0.974829	0.63187375	0.002762
F18	0.896471	−0.98079	0.994967	0.966964	0.53027348	0.003401
F19	0.896336	−0.98072	0.983412	0.964444	0.58287113	0.004922
Opti-Max	0.893369	**–0.97772**	0.977415	0.960530	0.51874445	−0.13430
Opti-Min	0.819541	−0.87222	0.915203	**0.917608**	0.84136923	16.60406

^a^ n ≤ 0.5 indicates Case I transport; Fickian diffusion; Higuchi release; t½ dependence, while *n* = 0.5-1 indicates Non-Fickian release; anomalous, the release is controlled by a combination of diffusion and polymer relaxation.

### 3.2. Response surface methodology for the optimization of MTC-loaded lipidic-nano-vesicular hybrids

#### 3.2.1. Estimation of the quantitative effects of the factors

The Statgraphics software was used to do multiple regression analysis with two-way ANOVA on the DLD batch data. Table 4 showed the estimated factors’ influence on the five ANOVA-generated results along with the corresponding *p*-values. If the effect deviates from zero and the *p*-value is less than 0.05, the influence of the factor is regarded as significant. A positive sign indicates a synergistic impact, whereas a negative sign indicates an antagonistic effect of the element.

**Table 4.** Estimated effects of factors and associated *p*-values for responses.

Factors	Y_1_		Y_2_		Y_3_		Y_4_		Y_5_	
	Factor effect	*P* value	Factor effect	*P* value	Factor effect	*P* value	Factor effect	*P* value	Factor effect	*P* value
X_1_	154.60	**0.037^a^**	5.30	0.366	21.21	**0.009^a^**	−7.30	0.121	−13.50	0.059
X_2_	37.32	0.498	−6.30	0.293	2.09	0.665	−14.10	**0.019^a^**	−17.30	**0.028^a^**
X_3_	77.83	**0.026^a^**	−3.98	0.163	4.17	0.106	−5.14	**0.037^a^**	−8.14	**0.024^a^**
X_4_	20.18	0.708	−3.30	0.561	5.77	0.268	6	0.182	15.40	**0.040^a^**
X_1_^2^	−4.72	0.921	−9.84	0.100	−6.59	0.173	−0.16	0.963	−5.16	0.322
X_1_X_2_	2.87	0.962	−1.63	0.794	−0.56	0.916	5.75	0.239	11.43	0.118
X_1_X_3_	−7.81	0.771	−7.68	**0.042^a^**	−1.63	0.507	−0.35	0.860	−4.53	0.153
X_1_X_4_	−44.83	0.469	−10.83	0.137	−1.35	0.801	−3.50	0.448	−4.68	0.462
X_2_^2^	−6.50	0.891	2.76	0.583	1.09	0.797	−1.36	0.701	−3.76	0.456
X_2_X_3_	−4.14	0.877	1.08	0.701	−1.47	0.548	5.35	**0.045^a^**	0.13	0.964
X_2_X_4_	4.63	0.938	−2.18	0.728	−2.09	0.698	1	0.822	5.18	0.419
X_3_^2^	65.80	0.214	−0.94	0.848	−2.01	0.640	0.038	0.991	2.34	0.636
X_3_X_4_	−5.16	0.847	−0.43	0.878	−0.46	0.849	0.80	0.689	4.025	0.193
X_4_^2^	8.90	0.851	−4.64	0.372	−1.67	0.697	−1.26	0.722	−10.66	0.080

X_1_ is the chitosan amount (mg), X_2_ is the cholesterol molar ratio concentration, X_3_ is the PEG 600 concentration percentage, X_4_ is the Tween 80 concentration percentage, X_1_X_2_, X_1_X_3_, X_1_X_4_, X_2_X_3_, X_2_X_4_, X_3_X_4_ are the interaction terms between the factors, X_1_^2^, X_2_^2^, X_3_^2^, X_4_^2^ are the quadratic terms of the factors, Y_1_ is the mean vesicle size (nm), Y_2_ is the zeta potential (mV), Y_3_ is the entrapment efficiency percentage, Y_4_ is the percentage of MTC initial release after 1 h and Y_5_ is the percentage of MTC cumulative release after 24 h.

^a^ Significant effect of factors on individual responses.

According to the results, there is a substantial synergistic relationship between the amount of chitosan (X_1_) and the mean vesicle size (Y_1_) and drug entrapment efficiency in nanovesicles (Y_3_), with *p* values of 0.037 and 0.009, respectively. In addition, with *p* values of 0.019 and 0.028, it was discovered that the cholesterol concentration (X_2_) has a substantial antagonistic influence on the initial release percentage of MTC after 1 h (Y_4_) and the cumulative release percentage after 24 h (Y_5_), respectively. Additionally, it was discovered that the PEG 600 concentration (X_3_) has a strong antagonistic effect on Y_4_ and Y_5_, with *p* values of 0.037 and 0.024, respectively, and a considerable synergistic effect on Y_1_ with a *p* value of 0.026. Moreover, a significant synergistic effect of Tween 80 concentration (X_4_) on Y_5_ was discovered, with a *p* value of 0.04. Furthermore, interaction term (X_1_X_3_) had an antagonistic effect on the prepared nanovesicles’ zeta potential (Y_2_) with a *p* value of 0.042, whereas interaction term (X_2_X_3_) had a synergistic effect on Y_4_ with a *p* value of 0.045.

#### 3.2.2. Mathematical modeling and statistical analysis of the experimental data

In order to create a mathematical model for each response, values for the mean vesicle size of nanovesicles (Y_1_), zeta potential (Y_2_), entrapment efficiency (Y_3_), the initial release percentage of MTC after 1 h (Y_4_), and the cumulative release percentage of MTC after 24 h (Y_5_) were evaluated. Equations (6)–(10) represent the outcomes of the multiple linear regression analysis for each response variable produced using the best fit approach.

(6) $$\text{Mean vesicle size }(Y_{1})=\;150.46+4.16\;X_{1}+145.92\;X_{2}-0.95\;X_{3}+1.28\;X_{4}-0.004\;X_{1}^{2}+0.23\;X_{1}X_{2}-0.01\;X_{1}X_{3}-0.06\;X_{1}X_{4}-52.01\;X_{2}^{2}-0.55\;X_{2}X_{3}+0.62\;X_{2}X_{4}+0.15\;X_{3}^{2}-0.01\;X_{3}X_{4}+0.02\;X_{4}^{2}$$

(7) $$\text{Zeta potential }(Y_{2})=\;43.09+0.97\;X_{1}-40.22\;X_{2}+0.09\;X_{3}+0.79\;X_{4}-0.008\;X_{1}^{2}-0.13\;X_{1}X_{2}-0.01\;X_{1}X_{3}-0.01\;X_{1}X_{4}+22.05\;X_{2}^{2}+0.14\;X_{2}X_{3}-0.29\;X_{2}X_{4}-0.002\;X_{3}^{2}-0.001\;X_{3}X_{4}-0.01\;X_{4}^{2}$$

(8) $$\text{Entrapment efficiency }(Y_{3})=\;57.04+0.78\;X_{1}-0.68\;X_{2}+0.49\;X_{3}+0.57\;X_{4}-0.005\;X_{1}^{2}-0.05\;X_{1}X_{2}-0.002\;X_{1}X_{3}-0.002\;X_{1}X_{4}+8.74\;X_{2}^{2}-0.2\;X_{2}X_{3}-0.28\;X_{2}X_{4}-0.005\;X_{3}^{2}-0.001\;X_{3}X_{4}-0.004\;X_{4}^{2}$$

(9) $$\text{Initial release }(Y_{4})=\;47.94-0.41\;X_{1}-36.06\;X_{2}-0.72\;X_{3}+0.27\;X_{4}-0.0001\;X_{1}^{2}+0.46\;X_{1}X_{2}-0.0005\;X_{1}X_{3}-0.005\;X_{1}X_{4}-10.89\;X_{2}^{2}+0.71\;X_{2}X_{3}+0.13\;X_{2}X_{4}+0.0001\;X_{3}^{2}+0.002\;X_{3}X_{4}-0.003\;X_{4}^{2}$$

 (10) $$\text{Cumulative release }(Y_{5})=\;114.17-0.57\;X_{1}-22.9\;X_{2}-0.42\;X_{3}+0.73\;X_{4}-0.004\;X_{1}^{2}+0.91\;X_{1}X_{2}-0.006\;X_{1}X_{3}-0.006\;X_{1}X_{4}-30.1\;X_{2}^{2}+0.2\;X_{2}X_{3}+0.69\;X_{2}X_{4}+0.005\;X_{3}^{2}+0.009\;X_{3}X_{4}-0.024\;X_{4}^{2}$$

#### 3.2.3. Effects on the mean vesicle size (Y_1_) and zeta potential (Y_2_)

**Figure 3.**
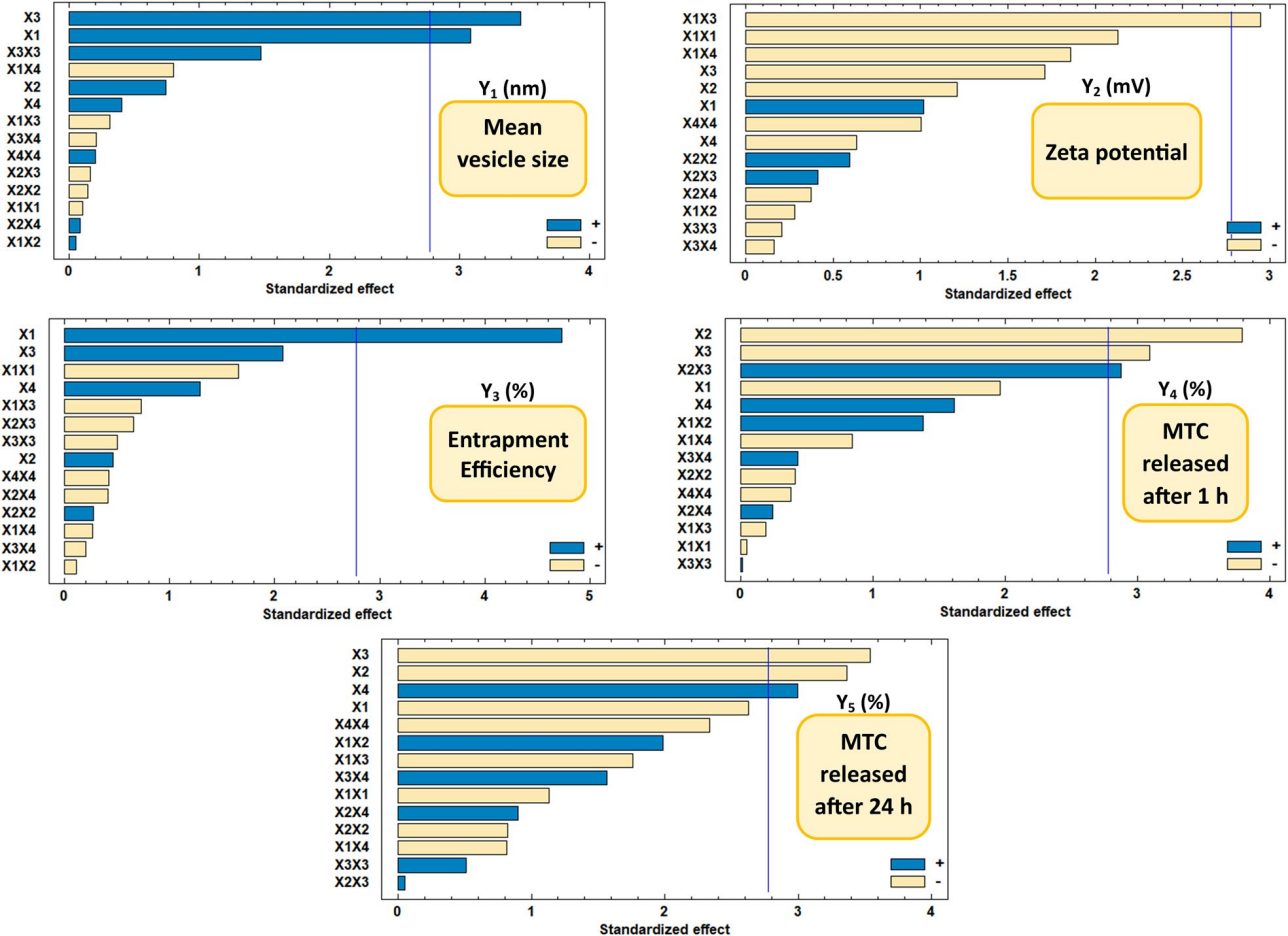
Pareto chart showing the standardized effects of factors on the observed responses (Y_1_–Y_5_).

**Figure 4.**
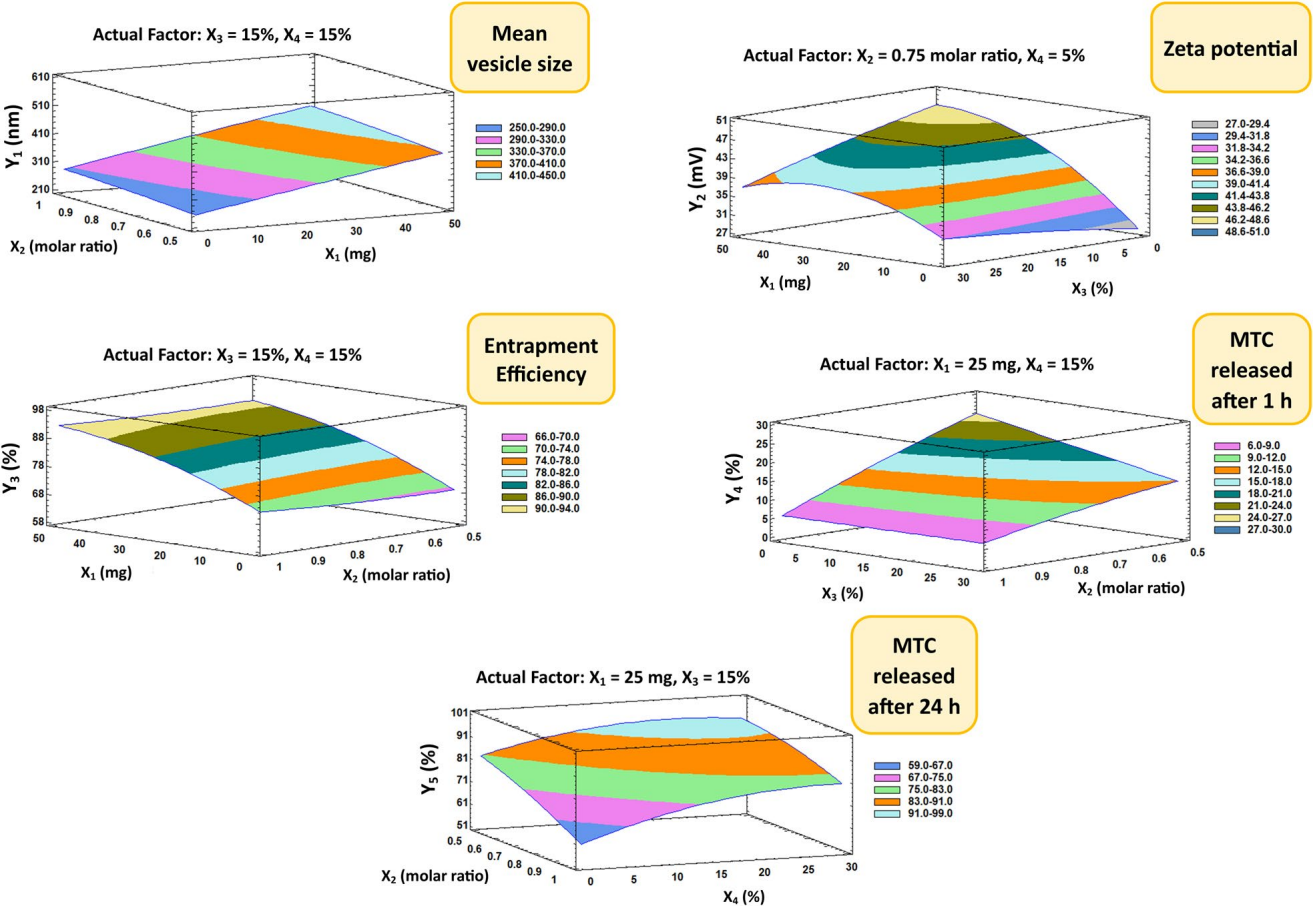
Contour-response surface plot showing the effects of factors on the observed responses (Y_1_–Y_5_).

The mean size of the nano-vesicular hybrids (Y_1_) ranged from 212.6 nm for F1 to 517.33 nm for F5 as shown in Table 2. It was found that PEG concentration (X_3_) has the main synergistic effect responsible for the difference in the vesicle size of the formulations as displayed in the pareto chart and the contour response surface plot of Y_1_ (Figures 3 and 4). At the constant level of X_1_, X_2_ and X_4_ in F7 and F8, the decrease of X_3_ from 30 to 0% was associated with the decrease of Y_1_ from 455.43 to 336.1 nm, respectively. On the other hand, the increase of X_3_ from 0 to 30% in F3 and F5, respectively, was evidenced with the increase of Y_1_ from 456.67 to 517.33 nm at fixed levels of all other factors. In addition, a direct/synergistic relationship was also found between the amount of chitosan (X_1_) and Y_1_. At the same level of X_2_, X_3_ and X_4_ in F16 and F17, the increase in X_1_ from 0 to 25 mg was associated with the increase of Y_1_ from 283.2 to 326.53 nm. Moreover, the decrease of X_1_ from 50 to 25 in F10 and F18, respectively, was evidenced with the decrease of Y_1_ from 437.8 to 304.68 nm at constant level of all other factors.

It is crucial to reconfirm that the zeta potential results (Y_2_) for any formulation with a negative value of zeta potential were applied as numerical values without considering the negative sign of the charge. This is done in order to correctly represent the mathematical correlations between the components and Y_2_ without any accidental data-misuse by the software. The zeta potential of the elaborated nano-vesicular formulations ranged from −27.3 mV (as a numerical value) for F1 to 47.8 mV for F8 (also as a numerical value) (Table 2). It was found that interaction term (X_1_X_3_) had an antagonistic influence on the prepared nanovesicles’ zeta potential, which were the only significant effect accountable for the alteration in the zeta potential values of DLD formulations (Figures 3 and 4).

#### 3.2.4. Effects on the EE% (Y_3_)

As mentioned in Table 2, the EE% (Y_3_) of the DLD nano-vesicular formulations was ranged from 59.6% in F1 to 93.85% in F12. It was detected that the chitosan amount (X_1_) is the main and only factor responsible for a significant influence on the percentage of EE of the elaborated formulations, which has been displayed in Pareto chart (Figure 3) and Contour-response surface plot (Figure 4) for Y_3_. A direct/synergistic association was found among X_1_ and the Y_3_. As it was noticed in F16 and F10, the increase of the chitosan amount from 0 to 50 mg was associated with the increase of Y_3_ from 69.18% to 90.39%, respectively, at the same level of other factors. It was detected too, that the decrease of chitosan amount from 50 to 25 mg, with making the levels of the other factors to be constant, would produce a decline in Y_3_ from 90.39% in F10 to 82.68% in F19.

#### 3.2.5. Effects on the initial (Y_4_) and the cumulative (Y_5_) MTC release

Table 2 revealed that F1 had the highest first MTC release rate (27.3%) and F5 had the lowest initial MTC release percentage (2.6%). Additionally, F1 released the highest cumulative MTC percentage (95,7%), whilst F5 released the lowest cumulative MTC percentage (51.9%). It was observed that both the cholesterol concentration (X_2_) and PEG 600 concentration (X_3_) have the main/antagonistic effect on the MTC release from the DLD nano-vesicular formulations, either after 1 h (Y_4_) assigned as initial release, or after 24 h (Y_5_) assigned as cumulative release. These outcomes were showed in Pareto charts (Figure 3) and Contour-response surface plot (Figure 4) for Y_4_ and Y_5_, respectively. In F9 and F6, as X_2_ molar ratio increased from 0.5 to 1 at fixed levels of all other factors, Y_4_ decreased from 19.4% to 5.3% and Y_5_ declined from 91.9% to 74.4%, respectively. On the other hand, the elevation in Y_4_ and Y_5_ values from 8.9% in F12 to 18.8% in F2 and from 72.4% in F12 to 81.5% in F2, respectively, was accompanied by the decline of X_3_ from 30 to zero percentage concentration while the levels of all other factors were fixed.

Furthermore, the Tween 80 concentration (X_4_) was found to affect significantly and synergistically on Y_5_ only, without having any other significant influence on other responses. Moreover, the interaction term (X_2_X_3_) was found to have a synergistic effect on Y_4_, without showing any significant influence on Y_5_ and the other responses as well.

### 3.3. Prediction and appraisal of Opti-Max/Opti-Min MTC–loaded PEG-T-Chito-Lip nano-vesicular formulations

Finding an optimized formula of nano-vesicular hybrids that meets our requirements for achieving both maximum/minimum goals in release after 1 h (initial) and release after 24 h (cumulative), as well as the minimal vesicle size, maximum zeta potential and entrapment efficiency, is made possible by multiple response optimization. In other words, both the initial and cumulative release responses have undergone two iterations of the multiple response optimization. One involved forecasting an optimized formulation with a rapid release target using a maximized-release goal (Opti-Max). The other was intended to reduce release in order to forecast an optimized formulation with a sustained or extended release goal (Opti-Min). In order to compromise between different responses and arrive at a combination of factor levels that magnify the desirability function, the final optimized experimental parameters were generated and assessed. A new two formulations were created in accordance with the projected model and analyzed for the responses in order to validate the dependability of the DLD results. The optimum value was 0.82 for the maximized release optimized formulation (Opti-Max), while the optimum value was 0.72 for the minimized release optimized formulation (Opti-Min).

The factors’ values for the Opti-Max and Opti-Min formulations namely; chitosan amount (mg), cholesterol concentration (molar ratio), PEG 600 concentration (%) and Tween 80 concentration (%), alongside the actual responses for mean vesicle size, zeta potential, entrapment efficiency, initial and cumulative release, were represented in Table 2. When the observed and anticipated responses values were compared, there were no significant residuals, showing that the used design was very effective for optimizing MTC nano-vesicular formulations (Mujtaba et al., 2014). These findings suggest that both optimized formulations achieved each goal’s targeted vesicle size, zeta potential, entrapment effectiveness, and drug release profile. Additionally, this result confirmed the consistency of the optimization procedure in reducing the vesicular size, increasing zeta potential, increasing entrapment efficiency, and simultaneously decreasing and increasing the amount of drug released. The *in-vitro* release profile of DLD optimized formulations, which served as the basis for the kinetics investigation (Table 3), were displayed in Figure 2.

The evaluation of the results of the mathematical modeling of the release mechanism (Table 3) revealed that the first order release mechanism represented the release of MTC from the Opti-Max optimized formulation (Nukulkit et al., 2014), confirming that the release is governed mainly by the high drug concentration (Gardouh et al., 2021; Kassem et al., 2017). On the other hand, the release of MTC from Opti-Min optimized formulation was followed by Higuchi diffusion mechanism, which evidenced the success of the formulation in controlling/extending the release pattern in accordance to the required goal (Desoqi et al., 2021; Megahed et al., 2022). The high (n) values (*n* > 0.5) for both formulations verified a non-Fickian diffusion mechanism for drug release, which suggests that the release is regulated by a combination of diffusion and polymer erosion or relaxation (Megahed et al., 2022; Shenoy et al., 2013).

### 3.4. Characterization of the Opti-Max/Opti-Min MTC–loaded PEG-T-Chito-Lip nano-vesicular formulations

#### 3.4.1. Assessment of the morphological outline

**Figure 5.**
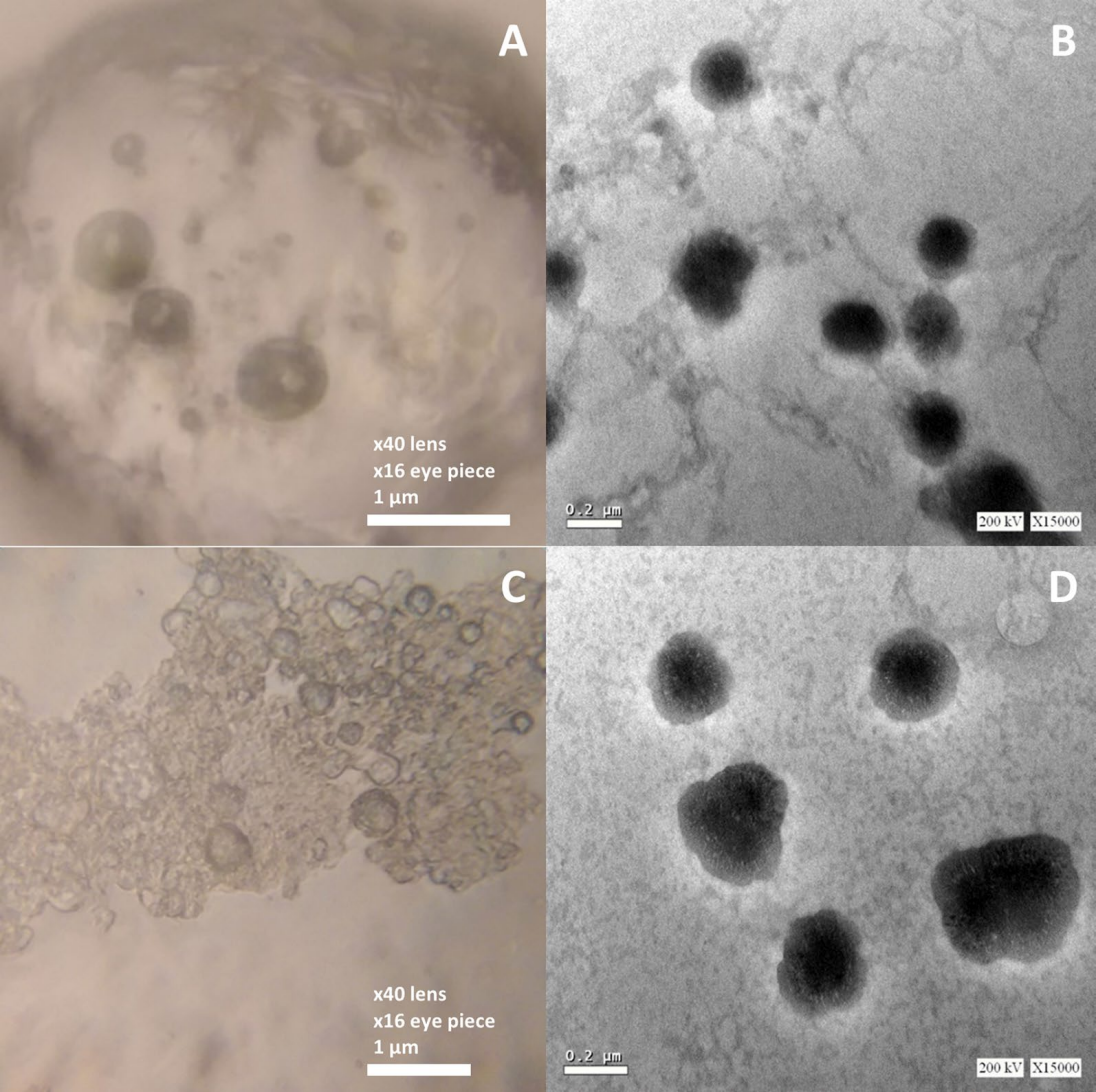
Light (left figures) and TEM (right figures) micrographs of the Opti-Max (A and B) and Opti-Min (C and D) MTC–loaded PEG-T-Chito-Lip nano-vesicular formulations. Abbreviations: MTC, metoclopramide hydrochloride; PEG-T-Chito-Lip nano-vesicular hybrid, PEGylated Tween 80–functionalized chitosan–lipidic nano-vesicular hybrid.

Photo microscopy was used to assess the morphological features of vesicles. Figure 5A and C displayed the morphology of the oval and regular rounded vesicles found in the two optimized formulations (Opti-Max and Opti-Min, respectively). Figure 5B and D showed the TEM photomicrographs of Opti-Max and Opti-Min formulations, respectively. Magnification set up used in TEM was ×15000, 200 kV.

The photomicrographs frequently revealed the vesicle’s outline and, to some extent, its inside structure. The oval and regularly rounded vesicles of the optimized formulations were visible in the electron micrographs, and their diameters ranged from 300 to 450 nm. Owing to the higher PEG 600 concentration in Opti-Min formulation, the PEGylation shielding was appeared in the TEM photomicrographs in multiple vesicular structures.

#### 3.4.2. Thermogravimetric analysis (TGA) and Differential Scanning Calorimetry (DSC)

**Figure 6.**
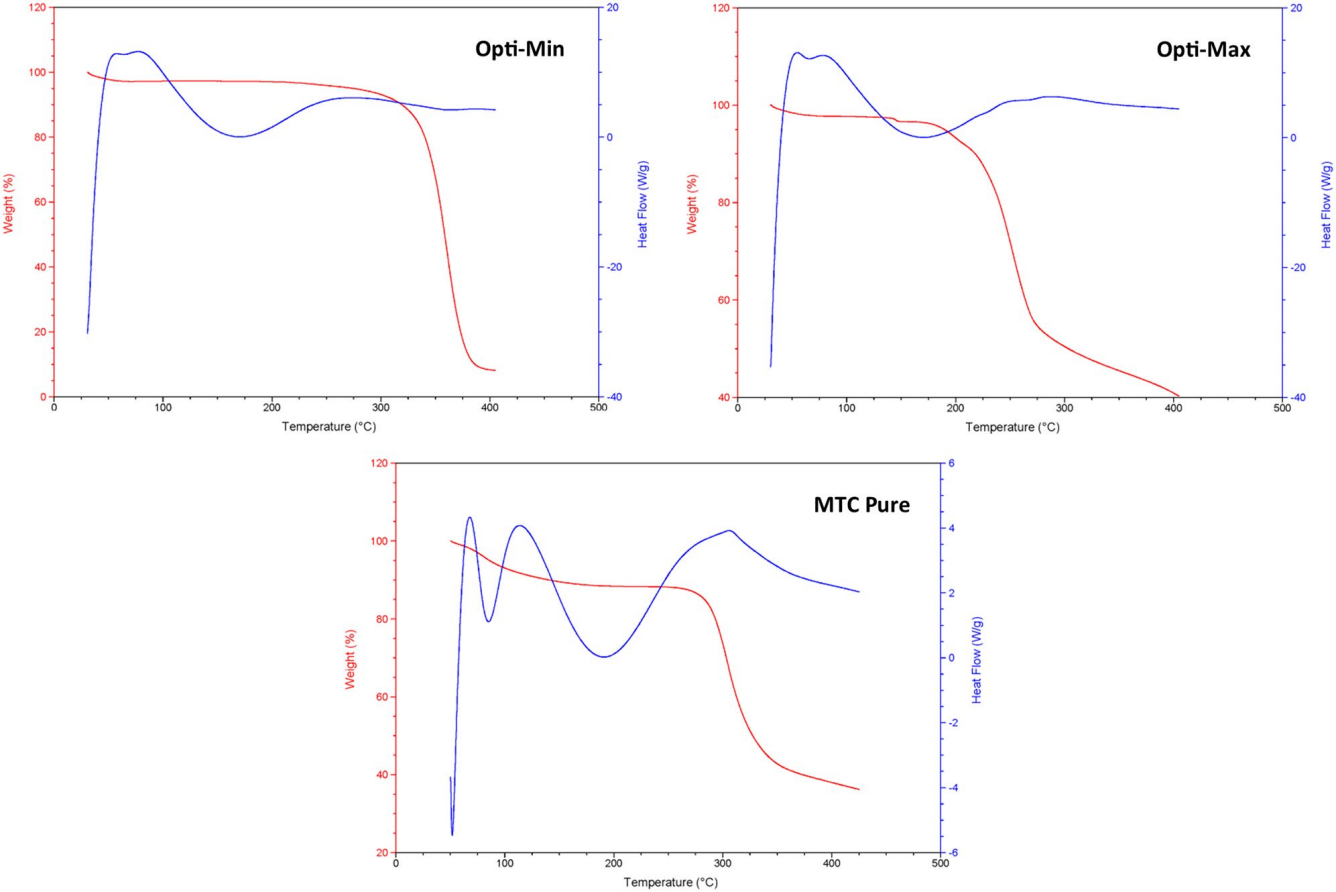
DSC/TGA thermograms of the pure MTC, Opti-Min and Opti-Max MTC–loaded PEG-T-Chito-Lip nano-vesicular formulations. Abbreviations: MTC, metoclopramide hydrochloride; PEG-T-Chito-Lip nano-vesicular hybrid, PEGylated Tween 80–functionalized chitosan–lipidic nano-vesicular hybrid.

The thermograms (Figure 6) and calorimetric values acquired for the two different optimized formulations (Opti-Max and Opti-Min) as well as the raw (pure) MTC were obtained. According to Figure 6, the loss of water molecules was the cause of the 11.11% weight loss of pure MTC that was seen in the TGA curve between 70 and 110 °C. The phase transition from the hydrated form to the anhydrous form occurred within this temperature range. According to the overlapping broad peak and following acute peak, this conversion entailed melting and dehydration, respectively (Nugraha & Uekusa, 2018). The melting of MTC was the subsequent thermal event, which was seen as a sudden endothermic peak at 186 °C with no accompanying weight loss. A total of 63.8% of the weight was lost after melting due to thermal breakdown, which occurred at temperatures between 275 and 425 °C.

The Opti-Max and Opti-Min MTC–loaded PEG-T-Chito-Lip nano-vesicular formulations’ thermograms demonstrate a shift in the transition temperature of each material used to formulate the optimized nanovesicles (Tween 80, Span 60, PEG 600, chitosan and cholesterol). Previous investigations provided the information on the thermograms, calorimetric parameters, and endothermic peaks for each ingredient (Gardouh et al., 2021; Kassem et al., 2017). The Span 60 transition temperature decreased in thermograms of both the optimized nano-vesicular formulations (Opti-Max and Opti-Min), and there was a definite peak extending for the optimized formulations at 161 °C. The distinctive endothermic peaks of the pure drug at 186 °C vanished from the DSC thermograms of the two optimized formulations as well. The enhanced encapsulation of MTC inside the nano-vesicular architecture of both optimized formulations can be supported by the notion that MTC interacts significantly with the nano-vesicular bilayer compositions (Gardouh et al., 2021; Kassem et al., 2017). All of these outcomes showed that the Opti-Max and Opti-Min MTC–loaded PEG-T-Chito-Lip nano-vesicular components interacted well to generate nano-vesicular bilayers, which improved the trapping of MTC in both optimized formulations.

### 3.5. Assessment of PK parameters of the intranasal dual-optimized MTC–loaded PEG-T-Chito-Lip nano-vesicular ISG formulation in plasma, brain and liver

**Figure 7.**
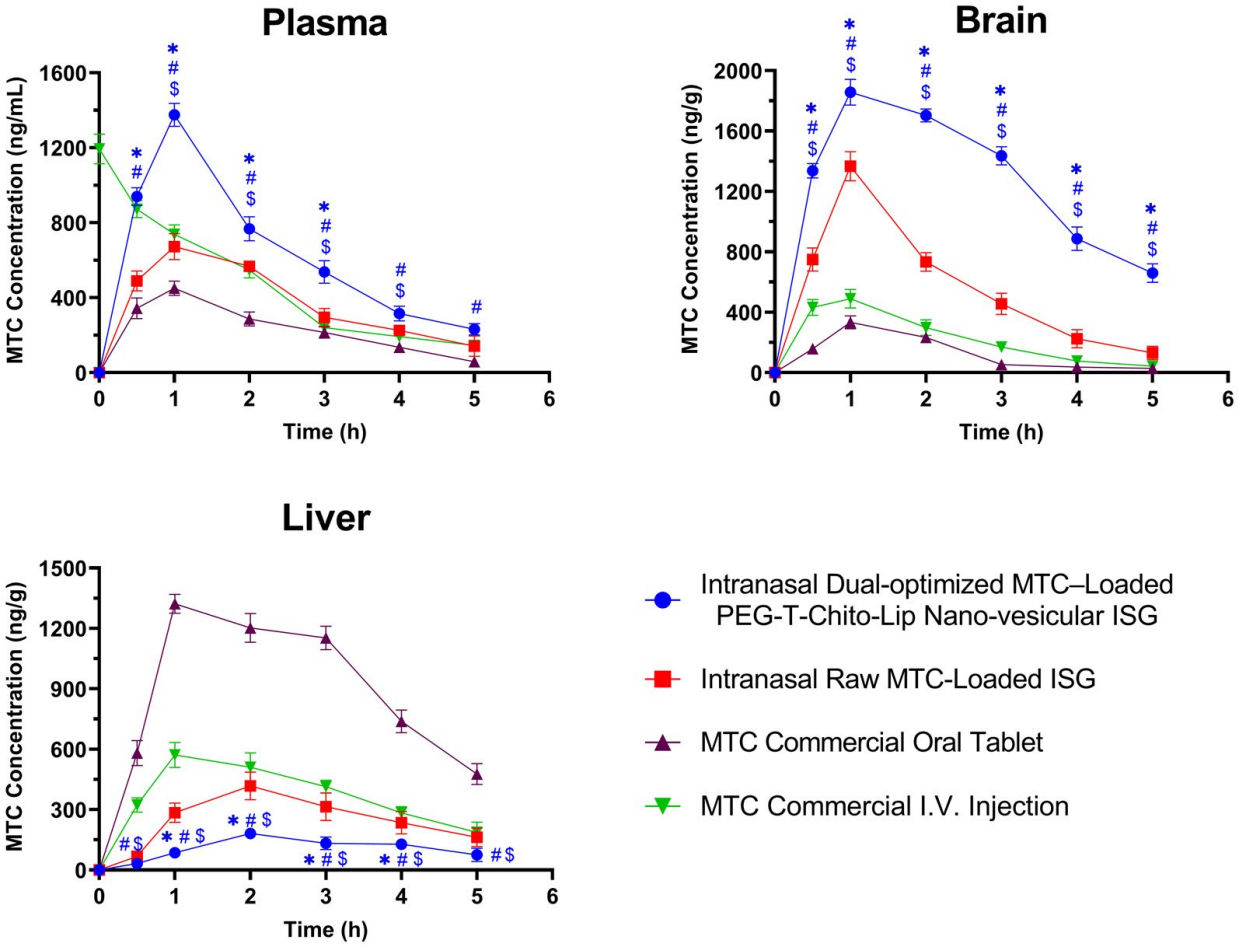
Plasma, brain and liver MTC concentration vs time profiles after administration of the intranasal dual-optimized MTC–loaded PEG-T-Chito-Lip nano-vesicular ISG, intranasal raw MTC-loaded ISG, MTC commercial oral tablet, and MTC commercial I.V. injection, in Sprague Dawley rats (*n* = 3). The dose of MTC in all formulations was equivalent to 3.5 mg/kg body weight. *, #, and $indicate *P* < 0.05 versus intranasal raw MTC-loaded ISG, MTC commercial oral tablet, and MTC commercial I.V. injection, respectively. Abbreviations: MTC, metoclopramide hydrochloride; PEG-T-Chito-Lip nano-vesicular hybrid, PEGylated Tween 80–functionalized chitosan–lipidic nano-vesicular hybrid; ISG, *in-situ* gel; I.V., intravenous.

Equal amounts of the Opti-Max and Opti-Min MTC–loaded PEG-T-Chito-Lip nano-vesicular formulations were combined and assigned as the dual-optimized MTC nano-vesicular formulation (with both maximized and minimized release goals and total MTC level of 0.5% w/v). Afterwards, the dual-optimized formula was furtherly incorporated within a Poloxamer 407 (22% w/v) and Carbopol 940 (0.5% w/v) *in-situ* gel (ISG) to form intranasal ISG for the evaluation of nose-to-brain delivery and *in-vivo* PK parameters. Figure 7 showed the plasma, brain, and liver concentration-time profiles for the intranasal dual-optimized MTC–loaded PEG-T-Chito-Lip nano-vesicular ISG formulation, the intranasal raw MTC-loaded ISG formulation, the MTC commercial oral tablet, and the MTC commercial I.V. injection. Table 5 provided a summary of the PK parameters’ values for MTC for all administered formulations.

**Table 5.** The PK parameters of MTC in the plasma, brain and liver after administration of the intranasal dual-optimized MTC–loaded PEG-T-Chito-Lip nano-vesicular ISG, intranasal raw MTC-loaded ISG, MTC commercial oral tablet, and MTC commercial I.V. injection, in Sprague Dawley rats (*n* = 3).

PK parameters of MTC in the plasma	Intranasal dual-optimized MTC–loaded nano-vesicular ISG		Intranasal raw MTC-loaded ISG		MTC commercial oral tablet		MTC commercial I.V. injection	
	Value	(± SD)	Value	(± SD)	Value	(± SD)	Value	(± SD)
$K_{\mathrm{el}}\;(h^{-1})$	0.44	0.012	0.41	0.16	0.62	0.21	0.29	0.15
t12 (h)	1.58	0.045	1.86	0.62	1.19	0.34	2.81	1.18
$T_{\max}\;(h)$	1	NA	1	NA	1	NA	–	–
$C_{\max}\;(\mathrm{ng}/\mathrm{ml})$	1375.33^a^^, b^	61.58	672.33^b, c^	69.21	450^c^	38.43	1193	79.57
${\mathrm{AUC}}_{0\text{-}t}\;(\mathrm{ng}/\mathrm{ml}\times h)$	3237.17^a^^, b, c^	220.84	1907.42^b, c^	94.49	1174^c^	82.64	2340.58	66.69
${\mathrm{AUC}}_{0\text{-}\infty }\;(\mathrm{ng}/\mathrm{ml}\times h)$	3765.06^a, b, c^	294.98	2320.11^b^	359.79	1278.91^c^	136.27	2949.92	333.16
${\mathrm{MRT}}_{0\text{-}\infty }\;(h)$	2.68	0.1	3.05	0.69	2.36	0.34	3.18	0.91
$V_{d}\;[\left(\mathrm{mg}/\mathrm{kg}\right)/\left(\mathrm{ng}/\mathrm{ml}\right)]$	2 х10^–3 a, b^	0.1 х10^–3^	3.98 х10^–3^	0.8 х10^–3^	4.7 х10^–3^	1.2 х10^–3^	4.7 х10^–3^	1.62 х10^–3^
Cl ([(mg/kg)/(ng/ml)]h)	9.3 х10^–4 a, b, c^	0.7 х10^–4^	15.3 х10^–4 b^	2.4 х10^–4^	2.8 х10^–3 c^	0.3 х10^–3^	1.2 х10^–3^	1.4 х10^–4^
PK parameters of MTC in the brain	Value	(± SD)	Value	(± SD)	Value	(± SD)	Value	(± SD)
$K_{\mathrm{el}}\;(h^{-1})$	0.38^c^	0.08	0.60^b^	0.12	0.33^c^	0.01	0.70	0.15
t12 (h)	1.86^c^	0.37	1.18^b^	0.21	2.10^c^	0.04	1.01	0.21
$T_{\max}\;(h)$	1	NA	1	NA	1	NA	0.83	0.29
$C_{\max}\;(\mathrm{ng}/g)$	1856.33^a, b, c^	86.09	1366.33^b, c^	97.34	333.67^c^	42.55	507.00	37.04
${\mathrm{AUC}}_{0\text{-}t}\;(\mathrm{ng}/g\times h)$	6416.92^a, b, c^	205.14	2878.25^b, c^	121.13	666.58^c^	88.01	1149.08	84.25
${\mathrm{AUC}}_{0\text{-}\infty }\;(\mathrm{ng}/g\times h)$	8206.36^a, b, c^	336.74	3109.06^b, c^	217.29	750.13^c^	92.46	1215.73	106.79
${\mathrm{AUC}}_{0\text{-}t/0\text{-}\infty }$	0.78^a, b, c^	0.05	0.93	0.03	0.89^c^	0.01	0.95	0.02
${\mathrm{MRT}}_{0\text{-}\infty }\;(h)$	3.47^a, b, c^	0.40	2.19	0.28	2.41^c^	0.10	1.99	0.20
$V_{d}\;[\left(\mathrm{mg}/\mathrm{kg}\right)/\left(\mathrm{ng}/g\right)]$	1.1 х10^–3 a, b, c^	0.2 х10^–3^	1.9 х10^–3 b, c^	0.2 х10^–3^	0.014^c^	2.02 х10^–3^	4.2 х10^–3^	0.7 х10^–3^
Cl ([(mg/kg)/(ng/g)]h)	0.4 х10^–3 a, b, c^	0.2 х10^–4^	1.1 х10^–3 b, c^	0.1 х10^–3^	0.01^c^	0.1 х10^–2^	0.3 х10^–2^	0.3 х10^–3^
The PK parameters of MTC in the liver	Value	(± SD)	Value	(± SD)	Value	(± SD)	Value	(± SD)
$K_{\mathrm{el}}\;(h^{-1})$	0.3	0.12	0.34	0.05	0.44	0.08	0.41	0.17
t12 (h)	2.55	0.83	2.08	0.36	1.60	0.31	1.87	0.68
$T_{\max}\;(h)$	2	NA	2	NA	1	NA	1	NA
$C_{\max}\;(\mathrm{ng}/g)$	181.33^a, b, c^	16.07	418.00^b, c^	68.51	1322.67^c^	47.09	571.00	62.23
${\mathrm{AUC}}_{0\text{-}t}\;(\mathrm{ng}/g\times h)$	559.58^a, b, c^	78.09	1295.75^b, c^	224.56	4613.50^c^	219.08	1891.75	111.98
${\mathrm{AUC}}_{0\text{-}\infty }\;(\mathrm{ng}/g\times h)$	862.19^a, b, c^	252.33	1799.69^b^	451.03	5732.17^c^	146.24	2430.83	310.64
${\mathrm{MRT}}_{0\text{-}\infty }\;(h)$	4.78	1.24	4.07	0.51	3.38	0.39	3.53	0.83
$V_{d}\;[\left(\mathrm{mg}/\mathrm{kg}\right)/\left(\mathrm{ng}/g\right)]$	1.5 х10^–2 a, b, c^	0.3 х10^–2^	0.6 х10^–2 b, c^	0.1 х10^–2^	0.1 х10^–2 c^	0.2 х10^–3^	0.4 х10^–2^	0.1 х10^–2^
Cl ([(mg/kg)/(ng/g)]h)	0.4 х10^–2 a, b, c^	0.1 х10^–2^	0.2 х10^–2 b^	0.5 х10^–3^	0.1 х10^–2 c^	0.2 х10^–4^	0.1 х10^–2^	0.2 х10^–3^

The dose of MTC in all formulations was equivalent to 3.5 mg/kg body weight.

^a^ Significant difference from values of intranasal raw MTC-loaded ISG with *p*-value <0.05.

^b^ Significant difference from values of o MTC commercial oral tablet with *p*-value <0.05.

^c^ Significant difference from values of o MTC commercial I.V. injection with *p*-value <0.05.

NA; not applicable.

Plasma concentration-time profile and PK parameters data showed that C_max_ of the intranasal dual-optimized MTC–loaded PEG-T-Chito-Lip nano-vesicular ISG was 1375.33 ng/ml at 1 h T_max_, which confirmed the significantly improved blood-C_max_ of the optimized formulation over 672.33 at 1 h (*P* = 0.0002), and 450 ng/ml at 1 h (*P* < 0.0001) for the intranasal raw MTC-loaded ISG and MTC commercial oral tablet, respectively (Figure 7 and Table 5). In addition, nearly all of the sampling points revealed a significant difference between the intranasal dual-optimized MTC–loaded PEG-T-Chito-Lip nano-vesicular ISG and all other studied groups (*P* < 0.05), indicating the remarkable improvement brought on by the intranasal delivery of MTC *via* the dual-optimized nano-vesicular ISG formulation, which was in agreement with many previous research works (Ahmed et al., 2019; Taweel et al., 2021).

The area under the curve (AUC) of the intranasal dual-optimized MTC–loaded PEG-T-Chito-Lip ISG also confirmed the significant bioavailability increase (superiority) of the dual-optimized intranasal system over all other MTC formulations (Table 5). The intranasal dual-optimized PEG-T-Chito-Lip nano-vesicular ISG have displayed an enhanced absorption profile of MTC in plasma (plasma-AUC_0-∞_) 1.639 and 2.9502 times over the plasma-AUC_0-∞_ of the intranasal raw MTC-loaded ISG and the MTC commercial oral tablet, respectively. In addition, the relative bioavailability of the intranasal dual-optimized formula to the MTC oral tablet (F_relative-oral MTC tablet_ = 295.02%) was found to be significantly higher (*P* = 0.0003) than the 180.9% F_relative-oral MTC tablet_ shown from the intranasal raw MTC-loaded ISG (Table 6). Interestingly, the absolute bioavailability of the MTC (F_absolute_) from the intranasal dual-optimized PEG-T-Chito-Lip nano-vesicular ISG was also significantly enhanced over the F_absolute_ of the intranasal raw MTC-loaded ISG and MTC commercial oral tablet (*P* = 0.004 and 0.0005, respectively), which were found to be 128.42%, 78.49%, and 43.5%, respectively (Table 6).

**Table 6.** The absolute and relative bioavailability parameters of MTC in the plasma after administration of the intranasal dual-optimized MTC–loaded PEG-T-Chito-Lip nano-vesicular ISG and intranasal raw MTC-loaded ISG in Sprague Dawley rats (*n* = 3).

Parameters (unit)	Intranasal dual-optimized MTC–loaded nano-vesicular ISG		Intranasal raw MTC-loaded ISG		MTC commercial oral tablet	
	Value	(± SD)	Value	(± SD)	Value	(± SD)
$F_{\mathrm{absolute}}\;(\%)$	128.42^a, b^	13.44	78.49^b^	5.48	43.5	3.72
$F_{\mathrm{relative}-\text{oral MTC tablet}}\;(\%)$	295.02^a^	10.84	180.92	12.66	–	–
$F_{\mathrm{relative}-\text{intranasal pure MTC}}\;(\%)$	163.9	17.93	–	–	–	–

The dose of MTC in all formulations was equivalent to 3.5 mg/kg body weight. MTC commercial I.V. injection and MTC commercial oral tablet were set as references for the calculation of the absolute and relative bioavailability, respectively.

^a^ Significant difference from values of intranasal raw MTC-loaded ISG with *p*-value <0.05.

^b^ Significant difference from values of o MTC commercial oral tablet with *p*-value <0.05.

Regarding brain MTC concentration-time profile, it showed that the profile of the intranasal dual-optimized MTC–loaded PEG-T-Chito-Lip nano-vesicular ISG was with a significant higher AUC with at least 10 times more than AUC of the MTC oral tablet (*P* < 0.0001), and almost 3 times more than AUC of the intranasal raw MTC-loaded ISG (*P* < 0.0001). The two-way ANOVA additionally verified a significant difference between the intranasal dual-optimized PEG-T-Chito-Lip nano-vesicular ISG and all other studied groups (*P* < 0.05) at each sampling point, supporting the dual-optimized formulation’s superiority over all other formulations in targeting the brain. Considering these findings, the intranasal dual-optimized MTC–loaded PEG-T-Chito-Lip nano-vesicular ISG can ensure effective and direct delivery of the loaded MTC to the brain by direct transport mechanisms (blood circulation passing through BBB) or *via* the olfactory area of the nasal mucosa (Sayyed et al., 2022). This can be rationalized from the interrelated effects of the positively-charged nanovesicles in enhancing brain delivery *via* Adsorptive-mediated transcytosis pathway, along with the stealthy behavior attained by using both PEG 600 and Tween 80 in formulating the dual-optimized formulation, for longer shelf life and hence, longer activity and overall improved brain-bioavailability (Claudio et al., 2016; Radwan, 2001).

The concentration-time profile for MTC in liver was also represented in Figure 7, which can be considered a good indication regarding the distribution of MTC to liver, and thus, extent of hepatic metabolism (Tian et al., 2019), and used to compare between the intranasal dual-optimized MTC–loaded PEG-T-Chito-Lip nano-vesicular ISG, intranasal raw MTC-loaded ISG, MTC commercial oral tablet, and MTC commercial I.V. injection. From the figure, it was revealed that the intranasal dual-optimized MTC–loaded PEG-T-Chito-Lip nano-vesicular ISG significantly had the lowest AUC versus all MTC formulations, particularly in comparison with the AUC of the MTC commercial oral tablet (*P* < 0.0001), as also mentioned in Table 5 (559.58 and 1891.75 ng.g ^− 1^.h ^− 1^, respectively). This confirms the success of the dual-optimized formulation in circumventing the hepatic circulation over the pure MTC-loaded ISG and the MTC commercial oral tablet.

The improved bioavailability and extended duration of the intranasal dual-optimized MTC–loaded PEG-T-Chito-Lip nano-vesicular ISG proved its success in accessing the brain (main site of action) and attaining the necessary longevity for sustained effect. It is believed that the optimized quantities of Tween 80, PEG 600, chitosan, and cholesterol used in formulating both Opti-Max and Opti-Min in the dual-optimized formulation, were successful in creating a dual diffusion mechanism, which produced the prompt/sustained profile for both the plasma and brain concentration-time curves. It is also can be related to the success of our intranasal dual-optimized formula in avoiding the extensive hepatic metabolism with much elevated MTC concentrations in the brain (Figure 7 and Table 5) as well as the significant lower clearance. The resultant enhanced absolute bioavailability (F > 1) was in agreement to many research works of other successful controlled/tailored-release dosage forms (Delrat et al., 2002; Jonkman et al., 1984; Schuppan et al., 1981), depending on the tailored release pattern and significant decreased total body clearance of drug vs the I.V. dosage form (*P* = 0.046). In addition, this significant enhancement can be also explained from another dual effect (as mentioned earlier), which is the chitosan-enhanced permeability to brain and Tween 80/PEG stealthy life-elongating effects. It is also witnessed that Tween 80 can give a synergistic effect with the utilized PEG and chitosan in bypassing BBB and yielding more efficient nano-vesicular brain delivery systems (Ahmed et al., 2019; Claudio et al., 2016). The great ability of the intranasal route to bypass both hepatic first pass metabolism and the insufficient intestinal absorption of MTC that occurs from the oral route is another fundamental cause for the improved bioavailability of MTC from the dual-optimized ISG intranasal formulation. This was consistent with other examples of intranasal delivery systems stated in a prior research studies (Zaki et al., 2007, 2006b).

### 3.6. Assessment of nose-to-brain delivery

**Figure 8.**
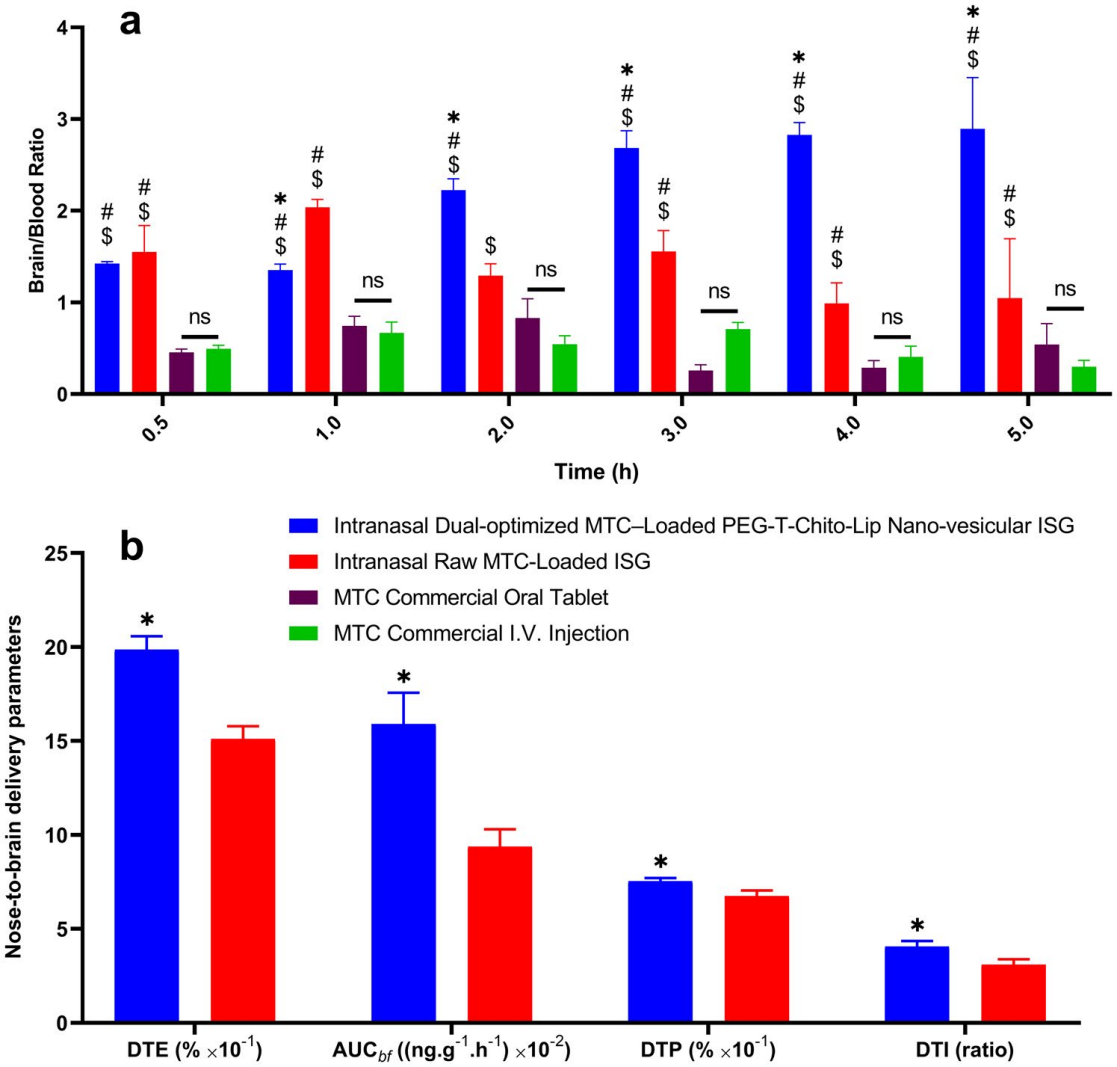
Evaluation of the nose-to-brain delivery of the dual-optimized MTC–loaded PEG-T-Chito-Lip nano-vesicular ISG formulation. a; MTC Brain/Blood ratio vs time of the intranasal dual-optimized MTC–loaded PEG-T-Chito-Lip nano-vesicular ISG, intranasal raw MTC-loaded ISG, MTC commercial oral tablet, and MTC commercial I.V. injection, in Sprague Dawley rats (*n* = 3). b; DTE%, DTP%, AUC*_bf_* and DTI of the intranasal dual-optimized MTC–loaded PEG-T-Chito-Lip nano-vesicular ISG and intranasal raw MTC-loaded ISG relative to the MTC commercial I.V. injection, in Sprague Dawley rats (*n* = 3). The dose of MTC in all formulations was equivalent to 3.5 mg/kg body weight. *, #, and $indicate *p* < 0.05 versus intranasal raw MTC-loaded ISG, MTC commercial oral tablet, and MTC commercial I.V. injection, respectively. Abbreviations: MTC, metoclopramide hydrochloride; PEG-T-Chito-Lip nano-vesicular hybrid, PEGylated Tween 80–functionalized chitosan–lipidic nano-vesicular hybrid; ISG, *in-situ* gel; I.V., intravenous; DTE: drug targeting efficiency; AUC*_bf_*: the AUC of the brain fraction; DTP: nose-to-brain direct transport percentage; DTI: drug targeting index.

The brain/blood ratio was calculated from plasma/brain concentration-time profiles of MTC after administration of the intranasal dual-optimized MTC–loaded PEG-T-Chito-Lip nano-vesicular ISG, intranasal raw MTC-loaded ISG, MTC commercial oral tablet, and MTC commercial I.V. injection in rats (Figure 8).

It was revealed that the brain/blood ratio for the intranasal dual-optimized MTC–loaded PEG-T-Chito-Lip nano-vesicular ISG was significantly different from all other MTC formulations (*P* < 0.05), especially in comparison with the oral commercial MTC tablet. This is another confirmation for the successful ability of the intranasal dual-optimized MTC–loaded PEG-T-Chito-Lip nano-vesicular ISG in targeting brain tissues along with the relative enhanced bioavailability of MTC.

DTE%, DTP%, AUC*_bf_* and DTI values were calculated for both the intranasal dual-optimized MTC–loaded PEG-T-Chito-Lip nano-vesicular ISG and the raw MTC-loaded ISG (Figure 8). The DTE % values, which measures the average partitioning time of the MTC between the brain and blood (Sayyed et al., 2022), were 198.55% and 151.03% for the intranasal dual-optimized MTC–loaded nano-vesicular ISG and the intranasal raw MTC-loaded ISG, respectively, suggesting that the dual-optimized formulation displayed significant faster and greater transport in comparison with the pure MTC-loaded ISG (*P* = 0.001).

On the other hand, DTP percentage of MTC (nose-to-brain direct transport through the olfactory and/or trigeminal nerve) was 67.43% and 75.26% for the intranasal raw MTC-loaded ISG and the intranasal MTC–loaded dual-optimized nano-vesicular ISG, respectively (*P* = 0.018). The higher DTE and DTP values for the intranasal dual-optimized ISG compared with those for the intranasal pure MTC ISG might explain the higher permeation of the dual-optimized nano-vesicular hybrids into the nasal mucosa, resulting in efficient brain delivery (Sayyed et al., 2022). Following nasal delivery, DTI measures the degree of MTC targeting to the brain; higher values indicate more effective MTC targeting of the brain (Sayyed et al., 2022).

As a result, the significant higher DTI values for the intranasal dual-optimized MTC–loaded PEG-T-Chito-Lip nano-vesicular ISG than for the intranasal raw MTC-loaded ISG (*P* = 0.016) revealed that the former supported more efficient drug transport to the brain.

### 3.7. Assessment of nasal mucosa irritancy upon application of the intranasal dual-optimized MTC–loaded PEG-T-Chito-Lip nano-vesicular ISG formulation

**Figure 9.**
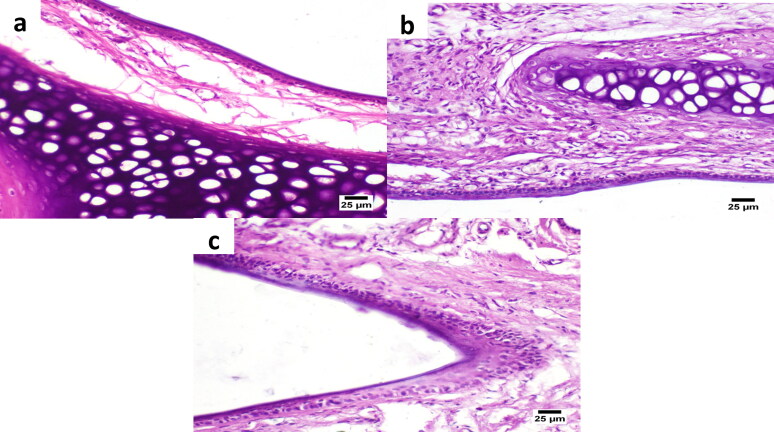
Histological images of the rat nasal mucosa after 5 h treatment with the intranasal dual-optimized MTC–loaded PEG-T-Chito-Lip nano-vesicular ISG (a) and intranasal raw MTC-loaded ISG (b), along with the normal untreated rat nasal mucosa (c) at time zero. The nasal cavity in all images showed apparently normal nasal mucosa (H&E).

The nasal mucosa histological photomicrographs after 5 h treatment with the intranasal dual-optimized MTC–loaded PEG-T-Chito-Lip nano-vesicular ISG are shown in Figure 9, which also displayed the histological photomicrographs of the nasal mucosa following 5 h treatment with the intranasal raw MTC-loaded ISG as well as the untreated (normal) nasal mucosa at time zero. On the treated nasal tissue, there are no indications of irritation or inflammation. Goblet cells and the ciliated respiratory epithelium have both been observed to appear normally. In light of this, and in good agreement with a prior study, the intranasal dual-optimized MTC–loaded PEG-T-Chito-Lip nano-vesicular ISG is deemed safe for usage when administered *via* the nose (Ahmed et al., 2019).

## 4. Conclusions

The MTC–loaded PEGylated Tween 80–functionalized chitosan–lipidic (PEG-T-Chito-Lip) nano-vesicular hybrids were successfully dual-optimized via Draper-Lin Design, which yielded two optimized formulations with different MTC release patterns. One with a rapid release behavior (Opti-Max) and the other with extended release profile (Opti-Min). Both Opti-Max and Opti-Min confirmed the efficient encapsulation of MTC *via* DSC-TGA analysis, as well as the nano-vesicular outline *via* light microscopy and TEM. The performance of the intranasal dual-optimized MTC–loaded PEG-T-Chito-Lip nano-vesicular ISG in avoiding the hepatic circulation over the intranasal pure MTC ISG and the MTC commercial oral tablet was evidenced along with its improved bioavailability, prolonged duration of efficacy, and brain delivery. When compared to the non-PEGylated non-Tween 80 intranasal raw MTC-loaded ISG, these findings showed a significant improvement in the PK parameters of MTC from the intranasal dual-optimized PEG-T-Chito-Lip nano-vesicular ISG. They also have confirmed the significance of the incorporation of Opti-Max with a high Tween 80 percentage along with Opti-Min with a high PEG 600 percentage. According to all prior results, the increased MTC bioavailability and heightened nose-to-brain delivery *via* the intranasal dual-optimized MTC–loaded PEG-T-Chito-Lip nano-vesicular ISG could promote patient convenience, adherence, and compliance. A future direction for more PK/PD/toxicokinetic studies to be performed for longer durations is from the high-priority research works to be conducted in our laboratory. In addition, comparative studies of single vs multiple dosing regimens are also of great interest. All this attention is to evaluate the capability of our dual-optimized formulation in maximizing patient compliance for much preferred clinical transition of the dual fast/long acting intranasal remediation, not only for MTC but also for other classes of drugs.

## CRediT authorship contribution statement

Saeed A. S. Al-Zuhairy: Methodology, Investigation, Data curation, Writing-original draft. Mahmoud H. Teaima: Conceptualization, Supervision, Validation, Writing—review & editing. Nabil A. Shoman: Resources, Validation, Writing—review & editing. Mohamed K. S. Mohamed: Resources, Validation, Writing—review & editing. Mohamed A. El-Nabarawi: Conceptualization, Project administration, Supervision, Formal analysis, Validation, Writing—review & editing. Hossam S. El-Sawy: Conceptualization, Supervision, Formal analysis, Validation, Writing-original draft, Writing—review & editing, Visualization. All authors have approved the submitted final version. All authors have read and agreed to the published version of the manuscript.
